# Global relationships between crop diversity and nutritional stability

**DOI:** 10.1038/s41467-021-25615-2

**Published:** 2021-09-07

**Authors:** Charlie C. Nicholson, Benjamin F. Emery, Meredith T. Niles

**Affiliations:** 1 Department of Biology, Lund University, Lund, Sweden; 2 Department of Entomology and Nematology, University of California, Davis, CA USA; 3 Vermont Complex Systems Center, University of Vermont, Burlington, VT USA; 4 Sandia National Laboratories, Albuquerque, NM USA; 5 Gund Institute for Environment, University of Vermont, Burlington, VT USA; 6 Food Systems Program, University of Vermont, Burlington, VT USA; 7 Department of Nutrition and Food Science, University of Vermont, Burlington, VT USA

**Keywords:** Biodiversity, Ecological networks, Sustainability, Agriculture

## Abstract

Nutritional stability – a food system’s capacity to provide sufficient nutrients despite disturbance – is an important, yet challenging to measure outcome of diversified agriculture. Using 55 years of data across 184 countries, we assemble 22,000 bipartite crop-nutrient networks to quantify nutritional stability by simulating crop and nutrient loss in a country, and assess its relationship to crop diversity across regions, over time and between imports versus in country production. We find a positive, saturating relationship between crop diversity and nutritional stability across countries, but also show that over time nutritional stability remained stagnant or decreased in all regions except Asia. These results are attributable to diminishing returns on crop diversity, with recent gains in crop diversity among crops with fewer nutrients, or with nutrients already in a country’s food system. Finally, imports are positively associated with crop diversity and nutritional stability, indicating that many countries’ nutritional stability is market exposed.

## Abstract (web summary)

Crop diversification could be important for food security. Here, using methods from network science, the authors find that a positive relationship between crop diversity and nutritional stability globally does not necessarily equate to improving nutritional stability in a given country.

## Introduction

Market volatility, land degradation, pests, and climate change make it increasingly difficult for agriculture to sustain a growing and healthy human population. In the face of these challenges, longstanding global- and national-scale policies seek to improve food security by increasing national production, leveraging international trade to counteract crop failures, and supporting technological innovation (e.g., precision irrigation, drought-resistant cultivars). These policies typically evaluate outcomes in terms of total yield or food calories; however, expanding commitments to nutrition-sensitive agriculture^1,2^ have recognized that calories do not equate to food security. There is now increasing focus on nutritional diversity, including diet diversity and the diversity of nutrients needed to sustain a balanced diet and lead an active, healthy life^3^.

With a growing focus on nutritional diversity, crop diversification is seen as a promising strategy to improve dietary diversity and nutritional status^4,5^. Crop diversity is a starting point toward ensuring more foods are available that could contribute to dietary diversity, but it is not itself a nutritional outcome. There is mixed evidence linking crop diversity to nutritional outcomes^6^, leading to calls for multilevel and systemic approaches that can measure the effect of agrobiodiversity on the provision of nutrients at different scales (e.g., village, region, and national)^7–9^. Potential nutrient adequacy^10^, one advancement toward this end, incorporates into a single metric the fraction of a population potentially nourished for all nutrients by all crops. However, neither this nor other approaches measures the ability of agriculture to produce nutritious food through space and time in the face of chronic disturbance and acute shocks^11^.

Quantifying nutritional stability—which we define as the capacity of a food system to provide nutrients despite disturbance—can aid effective coordination and implementation of resilience planning for nutrition-sensitive agriculture. Target 2 of the Sustainable Development Goals stresses connecting crop diversity, resilient farming systems, and nutritious diets as essential components of food security^12^. There are plausible mechanisms and demonstrated evidence for crop diversity contributing to human nutrition through the addition of key crops providing distinct nutrients^13–16^. Yet, the fragility of this diversity–function relationship is unknown because methods for tracking changes in stability have had limited application to food systems^17,18^.

**Fig. 1:**
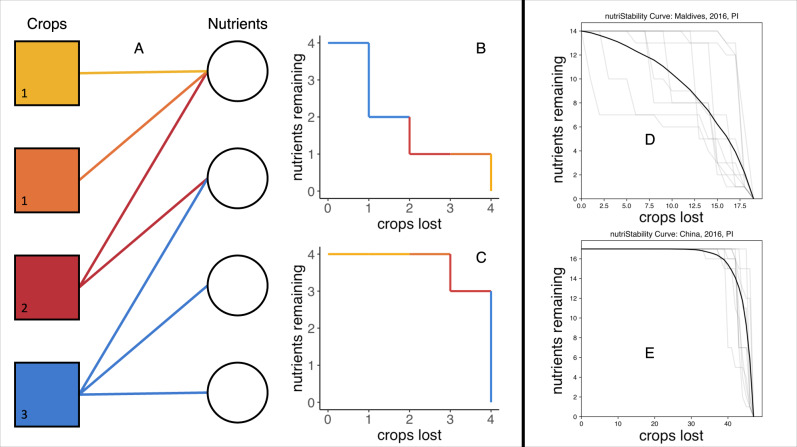
An approach for evaluating nutritional stability. We consider crops (boxes) and their nutrients (circles) a bipartite network (**A**). Numbers in the crop boxes indicate the number of nutritional links (crop degree). The stability of this crop-nutrient network can be gauged through assembling robustness curves (**B**, **C**), whereby as crops are eliminated, their constituent nutrients are lost. Species identity matters; crops with more nutritional links contribute more to overall stability. Networks with higher average degree (total links/total crops) are more robust to crop removal. Removal order also matters (**B** vs **C**); therefore permuted combinations of crop loss generate average robustness curves (see “Methods”). We use the area under this curve as our measure of nutritional stability (*R*_*N*_). In **D**, **E**, we are showing actual examples of multiple randomized removal sequences (light gray lines) and the average removal curve (black line). Crop-nutrient networks with few redundant connections are susceptible to crop removal and can experience rapid nutrient loss (e.g., Maldives; **D**), whereas countries with a diverse food supply are more robust (e.g., China; **E**). This unitless metric is generalizable across different levels of organization (e.g., individual, household, community, national food system).

Here we develop an approach that measures the extent to which crop diversity underpins nutritional stability over space and time. Our analytical framework (Fig. 1) links crops and their constituent nutrients into a bipartite network and then quantifies the effect on nutrient availability in a given country when crops are removed from the network, yielding a unitless metric that captures the robustness of different crop mixes for providing nutrients. This approach has characterized network robustness in diverse fields, from information systems^19^ to ecological networks^20^, but to our knowledge has never been applied to better understand the stability of nutrient availability in food systems. Importantly, crop species identity can affect overall network structure; a nutrient-rich crop will have greater degree (i.e., more connections within the network; Fig. 1A). A network with many nutrient-rich crops will possess high average crop degree (i.e., many crops that all have many nutrients) and is thus more robust to having individual crops lost from the network, because other crops likely contain the same nutrients. As with most biodiverse communities, species removal order can structure loss of system function^21,22^. We therefore generate a generalized “robustness curve”^23^ via permutation of removal sequence and derive our nutritional stability metric (*R*_*N*_) as the area under this curve (see “Methods”; Fig. 1D, E). We also perform two additional removal procedures: (1) crop removal from the most-to-least connected (i.e., remove crops with the most nutrients first), and (2) crop removal from the least-to-most connected (i.e., remove crops with the fewest nutrients first.

We apply this method to >22,000 crop-nutrient networks assembled using a global nutrient composition database^24^ and 55 years of Food and Agriculture Organization (FAO) food production data for 225 crops in 184 countries. Each country has its own network of crops and nutrients, which are used to quantify nutritional stability through our three removal procedures. In addition to nutritional stability, we calculate each network’s crop diversity (number of crops), nutrient diversity (number of nutrients available through crops), and average crop degree (number of nutrient links/number of crops). We evaluate patterns of nutritional stability and its relationship with crop diversity across countries, over time, and between two supply sources: food derived from production (*P*) and food from production and imports (PI). We then ask: (1) What is the relationship between crop diversity and nutritional stability? (2) How has crop diversity and nutritional stability changed over time in countries and regions? (3) What regional patterns underpin differences in nutritional stability? We find that, despite increases in crop diversity, nutritional stability has remained stagnant or decreased in all regions except Asia and that imports increase crop diversity and maintain nutritional stability.

## Results

### Crop diversity and nutritional stability

**Fig. 2:**
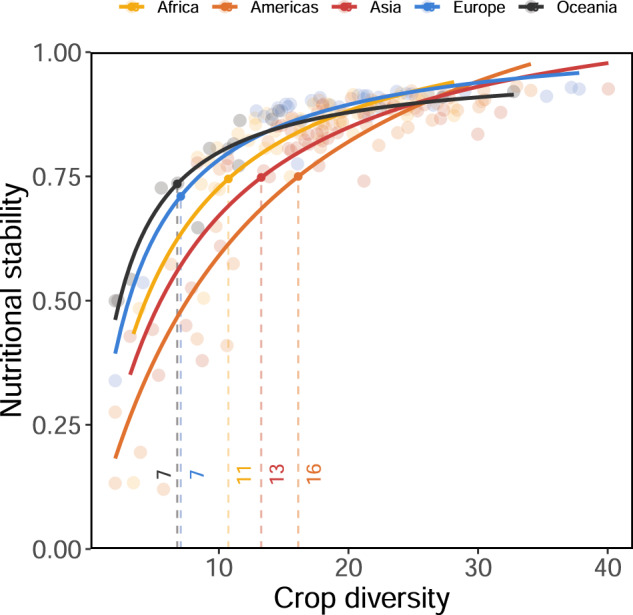
Nutritional stability increased non-linearly with crop diversity. Each region’s parameter values for this saturating function (*α* * *x*/(*β* + *x*)) differed significantly from each other (Supplementary Fig. 1 and Supplementary Table 1), indicating region-dependent responses to crop diversity. Each point is a country average across all years. Ordinates depict the level of crop diversity at which 75% of the maximum predicted nutritional stability will be achieved.

We find a non-linear relationship between crop diversity and nutritional stability that has regional variability (Fig. 2). Across countries, nutritional stability increases with crop richness at significantly (*P* < 0.05) different rates between regions, generally achieving a threshold at which additional crops do not provide significant improvements in nutritional stability (Fig. 2, Supplementary Fig. 1, and Supplementary Table 1). The average number of crops in a given country’s network varies between regions (range: 12–27), yet 83% of all networks possessed all 17 nutrients we examined here (Supplementary Fig. 2). We also found positive, non-linear relationships between crop diversity and nutrient diversity (i.e., more crops is associated with more nutrients available), and a linear relationship between nutrient diversity and nutritional stability (Supplementary Fig. 3). Across regions, gains in nutritional stability generally slow after crop-nutrient networks contain between 7 and 16 unique crops (Fig. 2). This suggests that there is a threshold for the extent to which increasing crop diversity in a region improves nutritional stability.

### Crop diversity trends

**Fig. 3:**
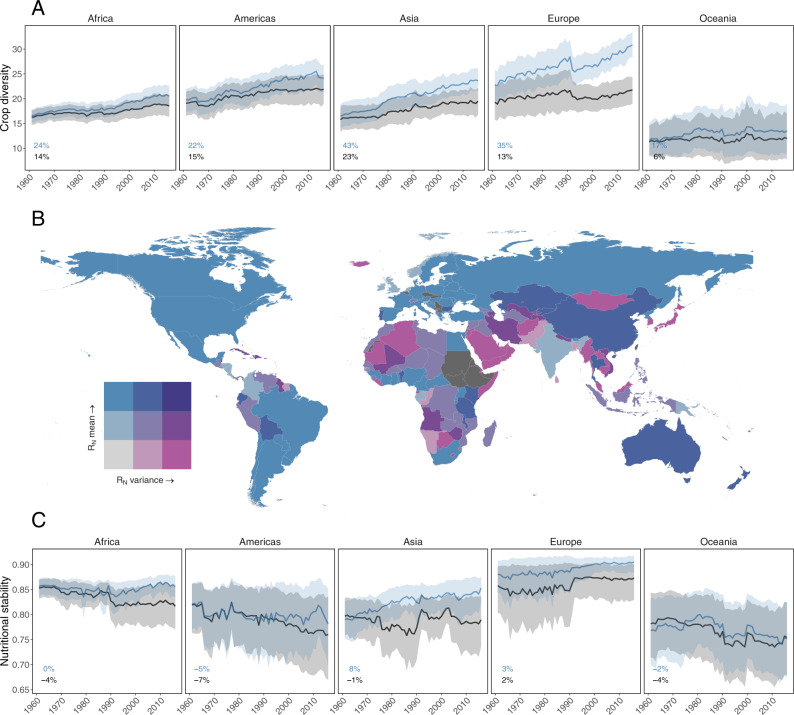
Global patterns and trends of crop diversity and nutritional stability. Crop diversity increased over the 1961–2016 period for production plus imports (blue) and production alone (black) (**A**). Nutritional stability (*R*_*N*_) varies over space (**B**) and through time (**C**). The bivariate choropleth map depicts *R*_*N*_ mean and variance for each country across years. For example, light purple countries experienced low average nutritional stability and high year-to-year variation. Countries filled with dark gray lacked sufficient data. Trends in *R*_*N*_ (**C**) varied between regions and depended on supply source (Table 1), with Africa and Asia exhibiting diverging responses for food supplies derived from production plus imports and production alone. Trend lines depict regional means ± 95% confidence intervals. The percentage change over the 55-year period for each region and different supply sources is in the lower left of each panel.

We find that crop diversity increased since 1961 for all regions except in Oceania (Fig. 3A and Supplementary Table 2). Importantly, there is significant variability across the remaining four regions, including the role of imports in driving crop diversity change over time. For example, in Asia and Europe imported crop diversity increased by 43 and 35% over the past 55 years, respectively. Europe also has the largest gap between imports and production, with the majority of crop diversity increases driven by imports. In other words, gains in crop diversity at the country level are not necessarily (and frequently not) the result of increased diversity of production within a country.

### Spatial variation in nutritional stability

**Fig. 4:**
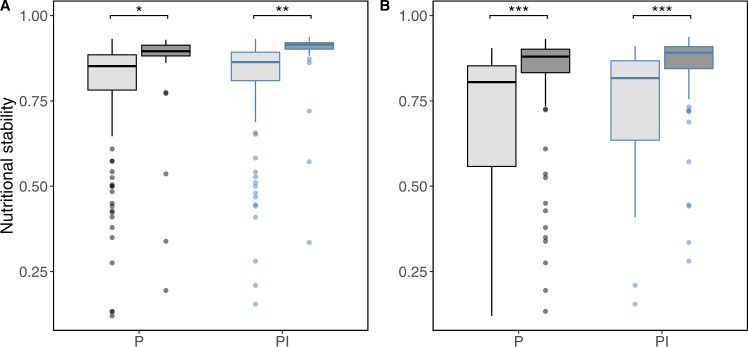
National factors related to nutritional stability. Developing states (**A**, light gray) and small island states (**B**, light gray) had lower nutritional stability for both supply sources. Each point is a country’s nutritional stability averaged across years (*n* = 184 countries, black: production alone; blue: production plus imports). Small island states are included in the developing states. Boxes show median and interquartile range (25th–75th percentiles); whiskers show values within 1.5 × IQR of the boxes. See Supplementary Table 3 for statistical comparisons, *P < 0.05, **P < 0.01, ***P < 0.001.

We find high spatial variation in nutritional stability between countries over time (Fig. 3B). For example, countries such as the United States, Brazil, and much of Europe (light blue areas) had high stability with low variability in that stability (i.e., lower variance in *R*_*N*_ over the period 1961–2016), indicating that there was a consistently high stability of nutrients available from crops in the country’s food supply. Conversely, other countries such as many in the Middle East, Southeast Asia, and Africa experienced low stability and large variance (light purple areas), indicating a highly variable and generally unstable supply of micronutrients available in these countries. Notably, small island developing states and low-income countries often experience low stability (Fig. 4 and Supplementary Table 3).

### Nutritional stability trends

Despite finding that crop diversity increased over time, we find that nutritional stability has remained stagnant or even decreased in all regions except Asia (Fig. 3C and Table 1). The direction of these trends did not change with different crop removal procedures (see “Methods”; Supplementary Fig. 4); however, nutritional stability was lower when crops were removed in the order of most-to-least connected (Supplementary Fig. 5). Furthermore, across all regions, any gain in nutritional stability at the regional level over the time period is exclusively associated with a region’s supply of nutrients available from production and imports, not regional production alone. For example, while Asia experienced an 8% increase in nutritional stability, this was from an increase in imports over the time period; nutritional stability from production alone actually declined by 1%. Conversely, Africa experienced a 4% decrease in nutritional stability derived from reductions in crop diversity at the production level alone, while import-based nutritional stability did not change between 1961 and 2016. European nutritional stability was both relatively high (compared to other regions) and stable across the time period. In the Americas and Oceania, we find production-based nutritional stability decreased by 7 and 4%, respectively. When looking at the nutritional stability of a region, it is therefore important to consider both production and trade.

**Table 1:** Nutritional stability trends over time.

	Nutritional stability				
	Africa	Americas	Asia	Europe	Oceania
Supply source	−1.360**	−1.391^.^	−2.197*	−0.394	−0.452
	(0.412)	(0.816)	(0.822)	(0.306)	(0.548)
Year	−0.0004**	−0.001**	−0.0003	0.0002*	−0.001**
	(0.0001)	(0.0003)	(0.0003)	(0.0001)	(0.0002)
Supply source × year	0.001***	0.001^.^	0.001**	0.0002	0.0002
	(0.0002)	(0.0004)	(0.0004)	(0.0002)	(0.0003)
Observations	5546	4217	4577	3076	1628
Log likelihood	13,648.020	9471.530	9456.794	7189.631	4561.369

Results are from region-specific linear mixed effects model with an interaction between supply source (production + imports vs. production alone) and year as fixed effects, country nested in source as random effects, and an autoregressive correlation structure to account for temporal autocorrelation. Values are model coefficients with standard error in parentheses.

*P < 0.05; **P < 0.01; ***P < 0.001.

**Fig. 5:**
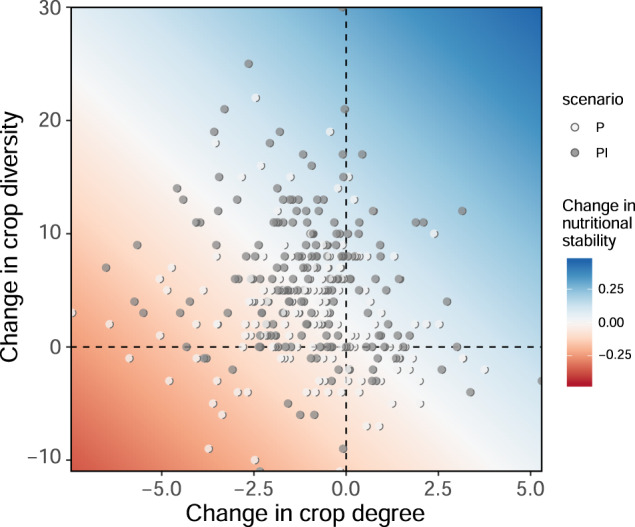
Change in crop diversity, degree, and nutritional stability. Each point depicts the difference between a country’s last (2016) and first annual record of crop diversity (i.e., number of crops) and crop degree (i.e., average number of nutrients connected to each crop) for production (*P*; white circles) and production plus imports (PI; gray circles). The response surface projects the expected change in nutritional stability as predicted by simple linear regression (*R*_*N*_ ~ diversity change + degree change; see Supplementary Table 7).

We present a seemingly counter-intuitive finding: for most regions crop diversity has increased yet nutritional stability is stagnant or decreasing. The saturating relationship between crop diversity and nutritional stability in part explains this, meaning that increasing diversity of crops does not necessarily add additional nutrients into a country’s food supply. However, changes in crop degree—the average number of nutrients provided by each crop in a network—clarify this incongruity further (Fig. 5). While crop diversity from production and imports increased for 72% of countries, 87% of these countries saw average crop degree decrease (Fig. 5, upper left quadrat), and this is associated with a decrease in nutritional stability. In fact, crop degree has declined in all regions over the 55-year period, regardless of supply source (Supplementary Fig. 6 and Supplementary Table 4). Thus, even though crop diversity has increased, there are diminishing returns on nutrient availability from adding more crops into a network, especially since the crops being added into networks appear to provide fewer links to nutrients not already in the food system. Nonetheless, it is worth noting that adding crops with few nutrients may be worthwhile if they provide links to “vulnerable” nutrients—those nutrients with few other links in the network.

## Discussion

Narrowing crop diversity in the world’s food supplies is a potential threat to food security^25^; however, there have been few empirical studies to link crop diversity to system-level nutritional measures, especially beyond dietary intake at the household level^9^. Here we develop a method to link crops to specific micronutrients using a network approach and assess the role of crop production and imports on nutritional stability outcomes in 184 countries between 1961 and 2016. Similar to other scholars^25,26^, we find that crop diversity has increased over time in many regions, but that in many cases these gains are due to imports. Despite this increase in crop diversity, nutritional stability has remained stagnant or decreased in all regions except Asia, a trend largely attributed to our finding that gains in crop diversity coincide with fewer new nutritional links in a given food system.

The general relationship between crop diversity and nutritional stability is contextualized by changes in crop degree and explains why stability does not mirror diversification trends. Improving crop diversity will always increase the size of the crop-nutrient network, but stability depends on the number and pattern of links within this network. As in other diversity–stability relationships functional identity matters, and declines in crop degree could reflect shifts toward networks with less nutrient-rich crops. For example, production-based crop diversity in Senegal increased by 29%, while crop degree dropped by 19% as the composition of its food supply shifted from staples (e.g., millet, groundnuts, sweet potatoes) to include less nutrient-dense crops (e.g., sugar cane, watermelon, cabbage). In light of on-going homogenization of crop diversity^26^, attaining the benefits of nutritional stability will require further understanding of the topology of crop-nutrient networks.

By considering both production and nutritional diversity, our approach advances the quantification of food system resilience—the capacity over time of a food system and its units at multiple levels, to provide sufficient, appropriate, and accessible food to all, in the face of various and even unforeseen disturbances^27^. Our results have many implications for our understanding of nutritional measures and their relationship to crop diversity. First, our work reaffirms the existing body of research demonstrating that crop diversity is important for agricultural resilience^11^, and it does so at a national scale. Previous work has examined patterns of crop or nutritional diversity at global scales^15,28^ or linked crop diversity and nutrition-relevant outcomes at the field or landscape levels^9^. Our work answers recent calls^8^ to explore crop diversity and nutrition-relevant outcomes at a larger scale through a country-level analysis and incorporates both production and imports, the latter of which has been significant for driving an increase in the types of crops available in a given country over time. To be clear, we are measuring the relationships of crop diversity to nutrients and their susceptibility to disturbance; we are not measuring nutritional outcomes such as dietary intake, dietary diversity, or other health-related outcomes that are the result of nutrition. Just as nutritional status cannot be determined from dietary intake alone, nutritional stability does not determine the availability, let alone utilization, of nutrients. This is a natural area to expand this work moving forward.

Second, our work establishes a functional relationship between crop diversity and nutritional stability. We suggest that this non-linear relationship has important implications for thinking about the types of crops grown or imported in a given region and how they ensure nutrient availability. A foundation shared by ecology and nutrition is that diversity can improve long-term functioning of complex biological systems^29,30^. Like other ecological diversity–resilience relationships, we observe that diversity loss can result in rapid loss of function^31^. In countries where diversity is already low, our results indicate that crop failures, either through production failure or an inability to import such crops, could lead to rapid reductions in nutrient availability within a country. Moreover, multiple failures of highly important regional crops, as might occur during a drought or other extreme events, could have catastrophic nutritional impact. Such countries are thus vulnerable to a variety of potential global challenges both ecological (e.g., climate change) and economic (e.g., trade wars).

Third, that nutritional stability is stagnant or decreased over time in all regions but Asia highlights that increasing crop diversity—at least at the national level—does not necessarily lead to more stability. Instead, the wide variability in nutritional stability across countries highlights clear vulnerabilities both across and within regions. Africa has the greatest inter-regional variability, demonstrating that in some cases neighboring countries have very different stabilities of crop nutrients in their food supply chain in any given year. This variability is likely driven by multiple factors including the capacity of a country to trade^32^, in country food availability as a result of war or political/social unrest^33–35^, or exposure to climate-induced disasters^36^.

Finally, the important role of imports in many regions highlights that crop diversity and nutritional stability are market exposed. While trade can positively affect food security^37^, it can also hinder nutrition efforts^38^ and could be a vulnerability if imports comprise a significant portion of nutritional stability for a given population. Countries with a high reliance on imports are thus subject to trade wars, market shifts, and price shocks that can occur for a variety of reasons^39^. Such countries may be more likely to experience increased variability in the future, especially as climate change is expected to affect agricultural production, markets, and trade^40^.

The use of these results could help inform high-level discussions within countries and regions about the key crops for a given place and their availability via import or domestic production. Scenario development using our metric could help target country-specific crop additions that would maximize nutritional stability. Our approach could also be used to identify potential tradeoffs in production and import outcomes, at least as it relates to the availability of a given amount of nutrients in a certain place. In the context of policy interventions, this system-level metric could be applied in panel-type designs to diagnose whether initiatives (e.g., promoting or increasing food production, trade and storage) at different scales of organization (e.g., household, community, national) will effectively promote food system resilience programs^41^.

Such potential applications also highlight the importance of identifying several caveats and important limitations. First, although we are addressing the nutrients available in a given country in a given time, we are not equating this with food security. This “availability” is only one component of food security, with access, utilization, and stability being other critical pillars. Thus, even though nutritional stability is generally high in most regions and remained stagnant (or increased in Asia), this does not mean that people are not food insecure. Adequate food and nutritional security comprises much more than the factors captured in our analysis, which provides a relative measure of nutrient availability not an absolute metric of adequacy. In the present study, we focused on nutrients available from crops, because animal-based products are rarely resolved to the species level and there is large interspecies variability in crop micronutrient composition. Animal-based products nonetheless play a critical role in providing some nutrients, thus there may be greater variability between countries when accounting for animal-based foods. There are also some methodological limitations. Crops are likely to vary in their loss susceptibility according to exogenous factors, such as market value or climate change vulnerability or pest pressure or simply abundance. In our current approach, all crops have equal removal probability; crop removal scenarios that account for these differential vulnerabilities is an exciting next step. Our current approach considers only nutrient presence or absence and may underestimate nutritional stability because ultimately the vulnerability of nutrient provision will also depend on how much of that nutrient is produced. Considering fractional crop loss or removal probabilities based on production levels could add realistic complexity in future analyses. Furthermore, complex system modeling of trade dynamics could explore to what extent import-based network re-orientation rescues nutritional stability by allowing for network rewiring via crop substitutability^42,43^. Finally, there are recognized shortcomings with the existing FAO data, especially in many low-income countries^44^. Nevertheless, to our knowledge, it is the best available data of its kind and scale available, so we utilize it knowing that there are many opportunities to improve this work moving forward.

Despite these caveats, this work advances a method to assess the relationship between crop diversity and nutrient availability globally over the past 55 years. Future research could expand this work in multiple ways by combining crop-nutrient availability data with nutritional intake data to better assess whether available nutrients in the supply chain are making their way into household consumption. This would more completely link crop diversity with food and nutritional security outcomes, rather than just food availability as this work has done. Furthermore, our network tolerance method could be advanced by exploring the importance of certain crops for a given country or region by considering non-random loss of crops. Finally, with climate change expected to affect the yields of many globally important crops^45^ and potentially cause multiple crop failures at once^36^, this type of analysis could advance our understanding of food system vulnerability to specific crop failures and provide guidance on climate adaptation efforts or crop diversification strategies to safeguard against climate change.

Resilience is now a central paradigm in many sectors—humanitarian aid, disaster risk reduction, climate change adaptation, social protection. Most analyses of resilience in food systems occur at household or community scales^17^ or focus on broader patterns of food production and distribution^18,39^. Erosion of biological diversity typically leads to loss of ecosystem functioning and services, likewise loss of crop diversity may to lead to potentially drastic shifts in nutritional stability. Together this and future analyses have the potential to direct the protection or restoration of crop diversity so as to best support nutrient availability that is stable to current and future challenges.

## Methods

### Experimental design and data sources

Crop composition information came from annual national-level food production data for years 1961–2016 obtained from the FAOSTAT database (FAO of the United Nations; http://fao-stat.fao.org/) for 201 nations that comprise >95% of the global population. This study advances nutritional stability as a method and concept using crop data. Animal-based products can play an important role in providing protein and micronutrients and for stability in case of shocks and anomalies^46^. The role of animal-based products is beyond the scope of this paper but will be of interest to investigate in further research. To explore the impact of international trade on nutritional stability, we created two food supply sources by evaluating national yield separately as its production (*P*) and production plus imports (PI) components. We linked annual national crop lists for each source with crop-specific nutrient data reported in the Global Expanded Nutrient Supply (GENuS) database (Smith et al.^24^), a global dataset of nutrient supply that reports the amount of nutrient available per 100 g of edible food for 23 nutrients across 225 food categories. This dataset provides estimates of nutrient availabilities but does not take into account variation in nutrient content resulting from climate, regional, management, or other exogenous variability. However, because we construct qualitative networks (i.e., based on nutrient presence or absence), these context-dependent shifts in nutrient content would not change the overall topology of networks. We focused on 17 nutrients (calcium, carbohydrates, copper, fiber, folate, iron, magnesium, niacin, phosphorous, potassium, protein, riboflavin, thiamin, vitamin A, vitamin B6, vitamin C, and zinc), omitting calories, dietary fiber, fat, saturated fatty acids, monounsaturated fatty acids, and polyunsaturated fatty acids. We are limited to the nutrients in the GENuS database and some globally important micronutrients are not included (e.g., Vitamin D, B12, iodine). Linking these two datasets results in 22,400 crop-nutrient networks, one for each country, year, and food supply source. However, the nutritional contribution of some foods is often negligible, yet because links in our bipartite crop-nutrient networks are treated as binary these would still be considered as contributing to stability. To overcome this minimum quantity problem^47^, we calculated the proportion daily value (PDV) per serving for each country for each food as: PDV = 100*N*(Prod/(Pop*DV)), where *N* is the grams of nutrient per 100 g of food (as provided by GENuS), Pop is a country’s population (United Nations, 2019), Prod is the amount of that food produced (FAOSTAT, 2019), and DV is the adult daily value of the food in grams (FDA, 2019). We used PDVs as link weights in each network and then define a threshold on the computed link weight, below which we consider the link as nonexistent. To do this, we compute the stability for each country in 2016 at values of the threshold cutoff ranging from 0 to 0.7. The mean of the stabilities decreases monotonically as a function of this cutoff, but the variance shows a plateau from 0 to 0.1 before decreasing. In order to magnify between-country variation while removing negligible links, we choose the threshold of 0.1, at the upper end of this plateau. Data availability varied among global regions and missing data values were more common in the early years of the dataset, we therefore removed countries that did not have at least 20 years reported (*N* = 17 countries), leaving 184 countries and 19,044 networks. For each of these networks, we calculated crop diversity (number of crops), nutrient diversity (number of nutrients), average crop degree (number of links/number of crops), and nutritional stability (see next section).

### Formalizing nutritional stability (*R*_*N*_)

Our algorithm processes networks through permutation of crop species removal order to generate a robustness curve and uses the area under this curve as a coefficient of stability. Consider the set of *M* crops *C* = {*c*_1_, · · ·, *c*_*M*_} and the set of *P* nutrients *N* = {*n*_1_, · · ·, *n*_*P*_}. We begin by constructing a bipartite graph, *G*, with left nodes consisting of the set *C* and right nodes consisting of the set *N*. Place an edge between *c*_*i*_ (the *i*th crop) and *n*_*j*_ (the *j*th nutrient) if crop *c*_*i*_ contains nutrient *n*_*j*_. Given a subset of crops *D* ⊂ *C*, define nbr(*D*) to be the neighborhood of *D*, meaning the subset of *N* that is connected by an edge to an element of *D*. Finally, given a set *A*, let |*A*| be the number of elements in *A* (thus |*N*(*D*)| is the number of elements that *D* is connected to). Now consider the following sampling procedure:

1. Initialize *C*^0^ = *C*
2. At step *k*, remove one randomly selected crop from *C*^*k*−1^, leaving the set *C*^*k*^, which contains *M* − *k* crops. Let *X* (*k*) = |nbr(*C*^*k*^)|, the number of nutrients connected to *C*^*k*^.
3. Repeat Step 2 *M* times (i.e., until there are no more crops left).

Thus, at each iteration the procedure described above removes a randomly selected crop and all of its edges from *G* and counts the number of nutrients that remain connected to rest of the graph. The procedure yields a trajectory *X*(*k*), with *k* ranging from 1 to *M*, which we will call a sample. Repeating this procedure multiple times yields multiple samples *X*_*i*_(*k*). We generate an average robustness curve from 1000 permutations of this procedure and take its integral as our coefficient of nutritional stability (*R*_*N*_). At each step *k* = 1,2,…,*M*, the height of the average curve at step *k* is the average of all the individual curve heights at step *k*. Finally, we normalize this curve so that max(|nbr(*C*^*k*^)|) = 1 and max(*k*) = 1 before finding the area under the curve for *R*_*N*_. It is worth noting that a Bayesian network approach could be used to efficiently derive a similar robustness metric via analytical solutions, rather than permutation^48,49^. Our unitless measure represents how robust a food system is to the sequential elimination of crops. It does not provide information on the identity of different crops or nutrients nor the utilization of nutrients or the nutrient adequacy of a selection of foods.

### Statistical analyses

To explore the relationship between crop diversity and nutritional stability (Fig. 2), we compared three non-linear functional forms (saturating, logarithmic, exponential) using non-linear mixed effects models with crop diversity as a fixed effect and country as a random effect. A saturating function (*α* * *x*/(*β* + *x*)) was selected based on Akaike information criterion (Supplementary Table 5) and used for subsequent analyses. We used separate non-linear models for each region to explore the relationship between nutritional stability and crop diversity averaged across years for each country. We test whether response curves for this relationship are different between regions by extracting individual country regression coefficients for the model parameters (asymptote, *α*; doubling time, *β*) from non-linear mixed effects models with country as a random effect. We then used linear models to compare parameter coefficient values between regions (Supplementary Fig. 1 and Supplementary Table 6). We conducted these analyses using production source crop information, but results did not qualitatively change if imports were included. To explore changes in crop diversity, nutritional stability, and average crop degree over time (Fig. 3A, C and Supplementary Fig. 2), we used separate linear mixed effects models for each region with an interaction between supply source and year as fixed effects, a random intercept for source nested in country, and a first-order autoregressive (AR1) correlation structure to account for the temporal dependency structure of our data for each country and source (corAR1(form = ~year|country/source). This term assumes correlations between any 2 years are simply the product of correlations between adjacent years—that is, constant exponential decay of correlation over time. Finally, to explore how nutritional stability differs between macroeconomic factors (Fig. 4; small island state status, development status) we averaged crop diversity across years for each country and used linear mixed effects models with an interaction between macroeconomic status and source as fixed effects and country as a random effect. We performed all analyses in R (v 3.6.1) using “nlme”^50^ and “lme4”^51^ for statistical modeling. The algorithm used to calculate nutritional stability was built in Python v 3.7.4.

### Reporting summary

Further information on research design is available in the Nature Research Reporting Summary linked to this article.

## Supplementary information

Supplementary Information

Peer Review File

Reporting Summary

## Data Availability

All data used in this manuscript is publicly available. The crop production quantity data used in this study are available from the FAO Stat database [http://www.fao.org/faostat/en/#data/QCL]. The GENuS nutrient composition data used in this study are available from the Harvard Dataverse [https://dataverse.harvard.edu/dataverse/GENuS]. The intake data used in this study are available from the USDA Branded Food Products Database [https://data.nal.usda.gov/dataset/usda-branded-food-products-database].

## Code availability

The methods used to calculate nutritional stability are available at the project repository: https://github.com/dbemerydt/nutriStability/tree/V1.0.1; code 10.5281/zenodo.5163361.
