# Supplementary material for: Global relationships between crop diversity and nutritional stability
Source: Nat Commun. 2021 Sep 7;12:5310. doi: 10.1038/s41467-021-25615-2 (PMC8423801; doi:10.1038/s41467-021-25615-2)
Supplement: Supplementary file 1 — Supplementary Information [file 41467_2021_25615_MOESM1_ESM.pdf]

Title

Global relationships between crop diversity and nutritional stability

Authors

Charlie C. Nicholson[1*], Benjamin F. Emery[2,3], Meredith T. Niles[4,5,6]

Affiliations

[1] Department of Entomology and Nematology, University of California, Davis, 95616, CA, USA
[2] Vermont Complex Systems Center, University of Vermont, Burlington, 05405, VT, USA
[3] Sandia National Laboratories, Albuquerque, NM, 87185, USA
[4] Gund Institute for Environment, University of Vermont, Burlington, 05405, VT, USA
[5] Food Systems Program, University of Vermont, Burlington, 05405, VT, USA
[6] Department of Nutrition and Food Science, University of Vermont, Burlington, 05405, VT, USA

Supplementary Materials

This file includes:

Supplementary Table 1. Parameter estimates for the non-linear relationship between crop diversity and nutritional stability.
Supplementary Table 2. Crop diversity trends over time.
Supplementary Table 3. Macroeconomic factors drive nutritional stability differences.
Supplementary Table 4. Crop degree trends over time.
Supplementary Table 5. Comparing the relationship between nutritional stability and crop diversity using three saturating model forms.
Supplementary Table 6. Comparing differences in parameter estimates.
Supplementary Table 7. Change in crop diversity, degree and nutritional stability
Supplementary Figure 1. Parameter estimates from non-linear mixed effects models relating crop diversity and nutritional stability.
Supplementary Figure 2. Distributions of crop and nutrient diversity for regions and supply sources.
Supplementary Figure 3. Nutrient diversity increases with crop diversity and is associated with greater nutritional stability.
Supplementary Figure 4. Trends of nutritional stability considering different crop removal procedures.
Supplementary Figure 5. Nutritional stability depends on removal order of crops.
Supplementary Figure 6. Average degree of crops in crop-nutrient networks decreased over time.

SUPPLEMENTARY TABLES

Supplementary Table 1. Parameter estimates for the non-linear relationship between crop diversity and nutritional stability. Curves fit with a saturating function ($\alpha *x/(\beta + x)$). This functional form was selected after multiple model comparison (Supplementary Table 5). Individual models were fit for each region. For details on regional differences see Figure S1 and Supplementary Table 6. Values are model coefficients with standard error in parentheses.

	Nutritional stability ~ $\alpha *x/(\beta + x)$				
	Africa	Americas	Asia	Europe	Oceania
α	1.097***	1.335***	1.131***	0.995***	0.979***
	(0.042)	(0.099)	(0.052)	(0.015)	(0.027)
β	4.965***	12.574***	6.738***	2.353***	2.263***
	(0.814)	(2.486)	(1.085)	(0.366)	(0.299)
Observations	50	39	46	33	15
Log Likelihood	67.642	39.932	56.713	71.664	26.597
Note:	. p<0.1; * p<0.05; ** p<0.01; *** p<0.001				

Supplementary Table 2. Crop diversity trends over time. Results are from region-specific linear mixed effects model with an interaction between source and year as fixed-effects, country nested in source as random effects and an autoregressive correlation structure (i.e., time-lag correlation) to account for temporal autocorrelation. Values are model coefficients with standard error in parentheses.

	Crop diversity				
	Africa	Americas	Asia	Europe	Oceania
Source	-59.466^{*}	-75.863^{*}	-130.076^{*}	-219.441^{***}	-31.948
	(23.739)	(33.737)	(50.347)	(36.733)	(35.314)
Year	0.047^{***}	0.060^{***}	0.071^{***}	0.047^{***}	0.018
	(0.008)	(0.012)	(0.018)	(0.013)	(0.013)
Source × Year	0.030^{*}	0.039^{*}	0.067^{**}	0.113^{***}	0.017
	(0.012)	(0.017)	(0.025)	(0.018)	(0.018)
Observations	5,546	4,217	4,577	3,076	1,628
Log Likelihood	-8,590.126	-7,130.420	-8,581.432	-5,601.669	-2,366.630

Note: . p<0.1; * p<0.05; ** p<0.01; *** p<0.001

Supplementary Table 3. Macroeconomic factors drive nutritional stability differences. Differences (estimate standard error in parentheses) in nutritional stability between (a) developing and non-developing countries and (b) small island developing states (SIDS). Results are from separate linear mixed effects models with an interaction between macroeconomic status and supply source as fixed-effects and country as a random effect.

(a)

source	contrast	estimate	SE	df	*t* ratio	*p* value
P	Developing - Non-developing	-0.0674	0.0265	363	-2.541	0.0115
PI	Developing - Non-developing	-0.0693	0.0265	363	-2.612	0.0094

(b)

source	contrast	estimate	SE	df	*t* ratio	*p* value
P	Non-SIDS - SIDS	0.133	0.0257	363	5.172	< 0.0001
PI	Non-SIDS - SIDS	0.129	0.0255	363	5.083	< 0.0001

Supplementary Table 4. Crop degree trends over time. Results are from region-specific linear mixed effects model with an interaction between source and year as fixed-effects, country nested in source as random effects and an autoregressive correlation structure (i.e., time-lag correlation) to account for temporal autocorrelation. Values are model coefficients with standard error in parentheses.

	Crop degree				
	Africa	Americas	Asia	Europe	Oceania
Source	-4.168	-8.645	-0.841	35.491***	-9.692
	(7.645)	(9.645)	(10.455)	(5.733)	(16.069)
Year	-0.022***	-0.024***	-0.020***	-0.011***	-0.015**
	(0.003)	(0.003)	(0.004)	(0.002)	(0.006)
Source × Year	0.002	0.004	0.0005	-0.018***	0.005
	(0.004)	(0.005)	(0.005)	(0.003)	(0.008)
Observations	5,546	4,217	4,577	3,076	1,628
Log Likelihood	-4,488.510	-2,567.151	-3,741.008	-2,694.267	-1,716.429
Note:		. p<0.1; * p<0.05; ** p<0.01; *** p<0.001			

Supplementary Table 5. Comparing the relationship between nutritional stability and crop diversity using three saturating model forms. Based on AIC scores the saturating function $\alpha * x/(\beta + x)$ was used in subsequent analyses (Fig. 2; Supplementary Table 1 & Supplementary Table 6). Values are model coefficients with standard error in parentheses.

	Nutritional stability		
	$\alpha + \beta * \log(x)$	$\alpha * x/(\beta + x)$	$\alpha * \exp(\beta * x)$
β	0.161***	5.126***	0.017***
	(0.004)	(0.462)	(0.001)
α	0.234***	1.085***	0.592***
	(0.002)	(0.023)	(0.017)
Observations	183	183	183
Log Likelihood	184.529	200.093	125.840
AIC	-359.059	-390.186	-241.679
Note:	. p<0.1; * p<0.05; ** p<0.01; *** p<0.001		

Supplementary Table 6. Comparing differences in parameter estimates. Parameter values for region-specific relationship between nutritional stability and crop diversity (Africa is reference contrast). Curves fit with a saturating function ($\alpha * x / (\beta + x)$) via non-linear mixed effects models (see Methods) and coefficient values were extracted from random effects for each country. Values are model coefficients with standard error in parentheses.

	Saturating function parameter	
	α	β
Americas	0.238***	7.619***
	(0.00000)	(0.100)
Asia	0.034***	1.762***
	(0.00000)	(0.095)
Europe	-0.102***	-2.602***
	(0.00000)	(0.105)
Oceania	-0.118***	-2.692***
	(0.00000)	(0.137)
Observations	183	183
Note:	. p<0.1; * p<0.05; ** p<0.01; *** p<0.001	

Supplementary Table 7. Change in crop diversity, degree and nutritional stability. Results are from a linear model testing whether change in crop diversity and degree explain variation in nutritional stability change (R_N ~ diversity change + degree change).

	Estimate	Std. Error	t value	P value
Intercept	-0.016	0.005	-3.290	0.001
Crop degree change	0.031	0.002	15.280	<0.001
Crop diversity change	0.010	0.001	16.740	<0.001

SUPPLEMENTARY FIGURES

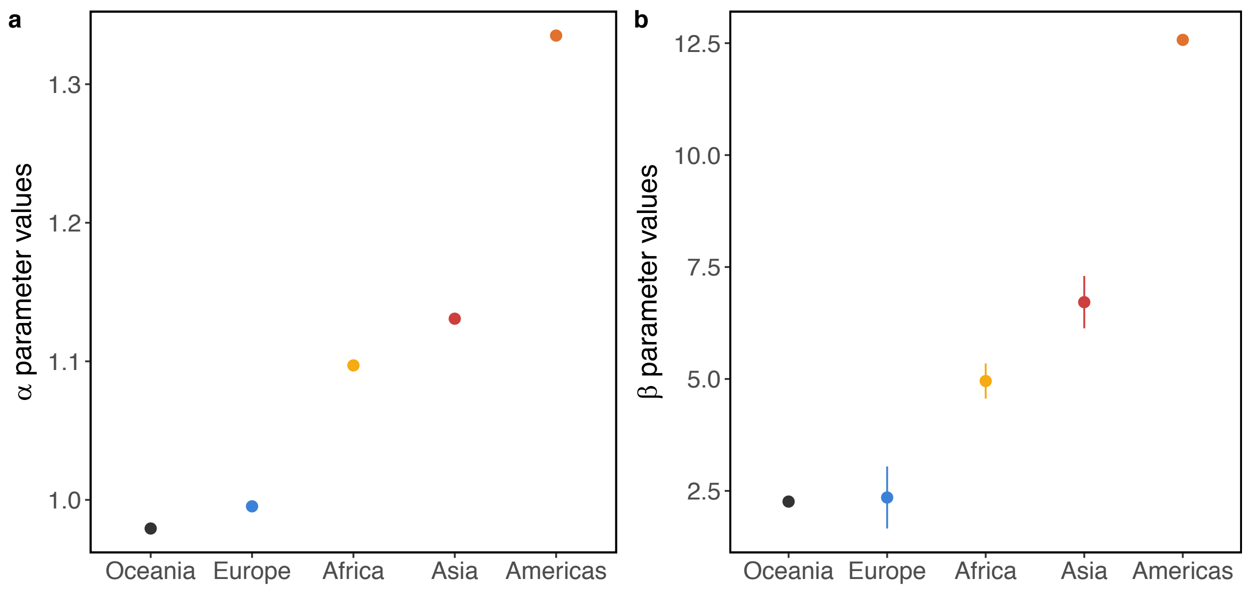

Supplementary Figure 1. Parameter estimates from non-linear mixed effects models relating crop diversity and nutritional stability. Curves fit with a saturating function ($\alpha * x/(\beta + x)$) via non-linear mixed effects models (Supplementary Table 6; see Methods) and coefficient values were extracted from random effects for each country. Points depict the average ± sd across countries for the α parameter (A) and β parameter (B).

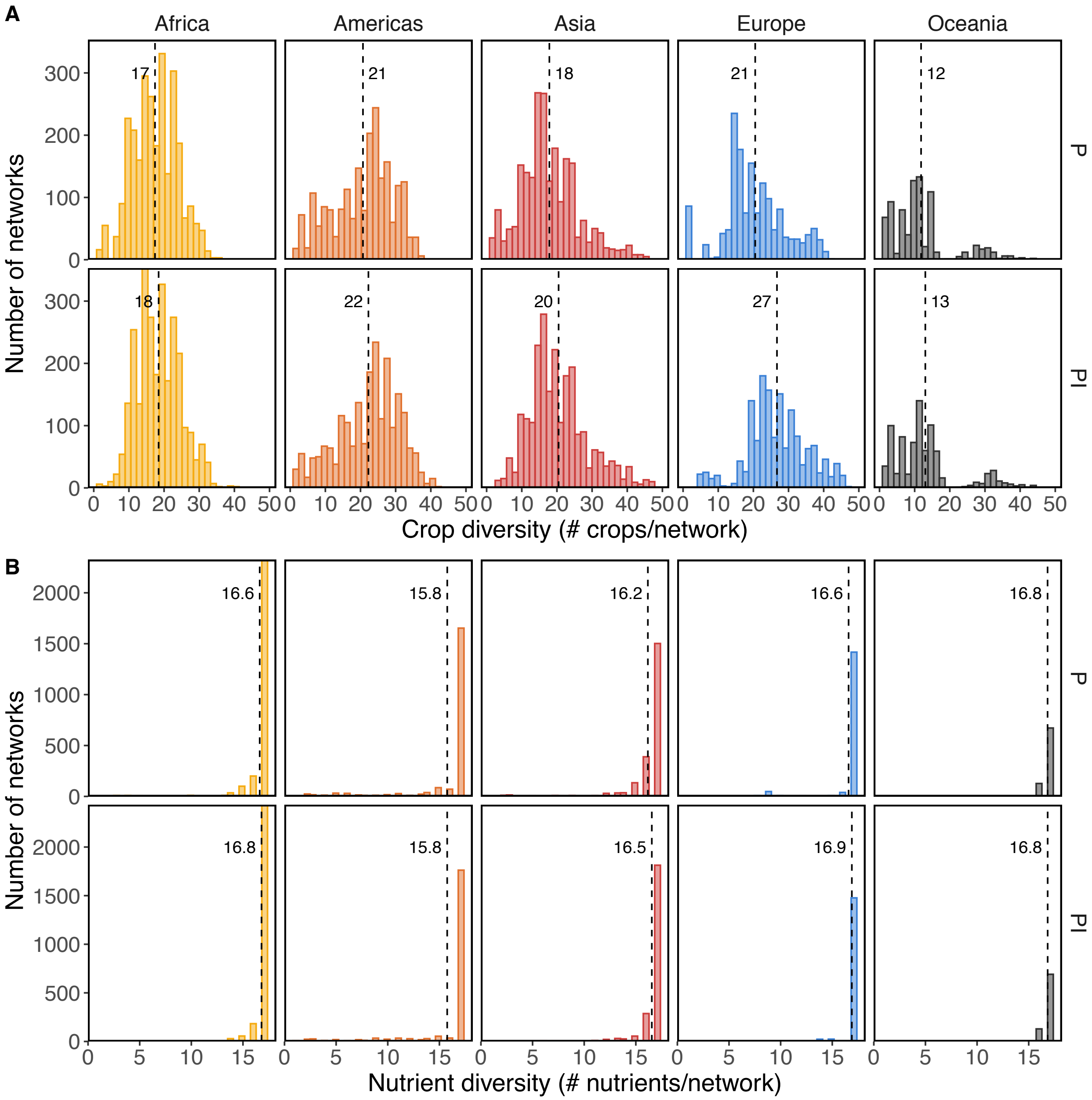

Supplementary Figure 2. Distributions of crop and nutrient diversity for regions and supply sources. Each bar depicts a region's number of networks for separate country-year combinations belonging to a specific levels of crop diversity (A) or nutrient diversity (B) for both production (P; top rows) and production and imports (PI; bottom rows) sources. Average values across countries and years are provided and depicted by the dashed vertical line. Crop diversity could be comprised of 225 different FAO food balance crop commodities. There are 17 micro-nutrients available in the GeNUS dataset that we analyzed (calories, fats, water, ash and refuse were not included). Over 83% of all crop nutrient networks (N = 19044) possessed all 17 micro-nutrients that we analyzed here.

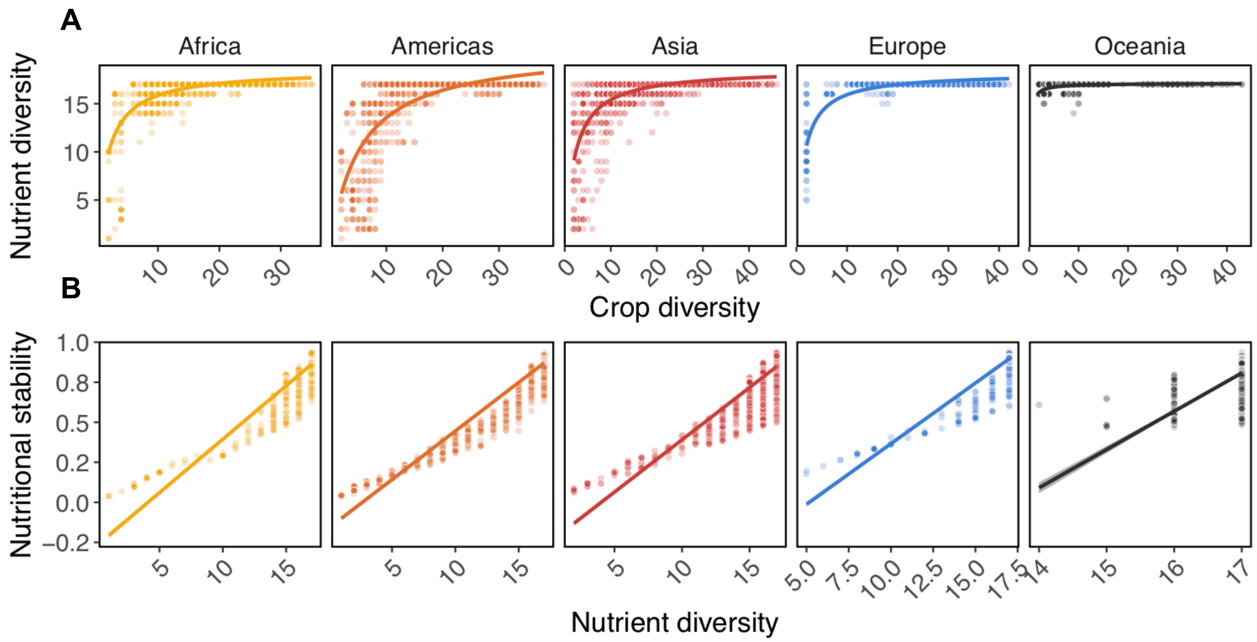

Supplementary Figure 3. Nutrient diversity increases with crop diversity and is associated with greater nutritional stability. Each point represents the crop diversity, nutrient diversity or nutritional stability from a country's crop-nutrient network in a given year. Non-linear relationships were fitted with same saturating function ($\alpha * x/(\beta + x)$) as in the main text.

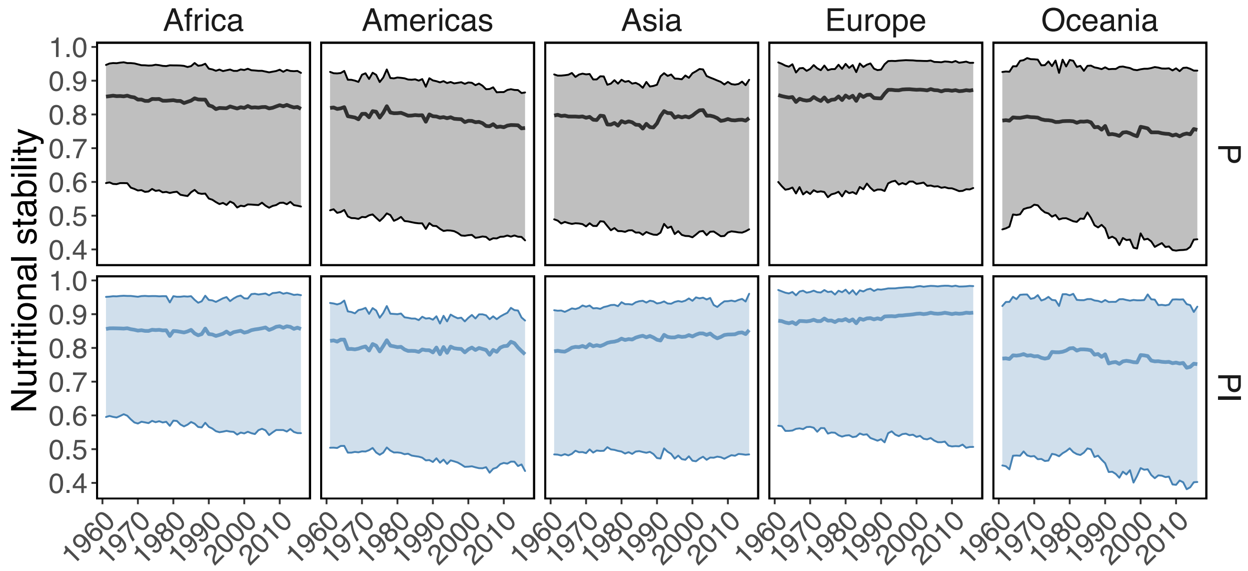

Supplementary Figure 4. Trends of nutritional stability considering different crop removal procedures. Nutritional stability (R_N) can be calculated different ways by changing the removal sequence of crops. In the main manuscript we report R_N values based on randomized crops loss. We also ordered crop loss from most to least connected crops (i.e. from those containing the most nutrients to those containing the fewest), and vice versa. Here we show trends in randomized R_N (solid middle line) with an upper bound derived from least-to-most removal and a lower bound derived from most-to-least removal for both production (P; top row) and production + imports (PI; bottom row) sources.

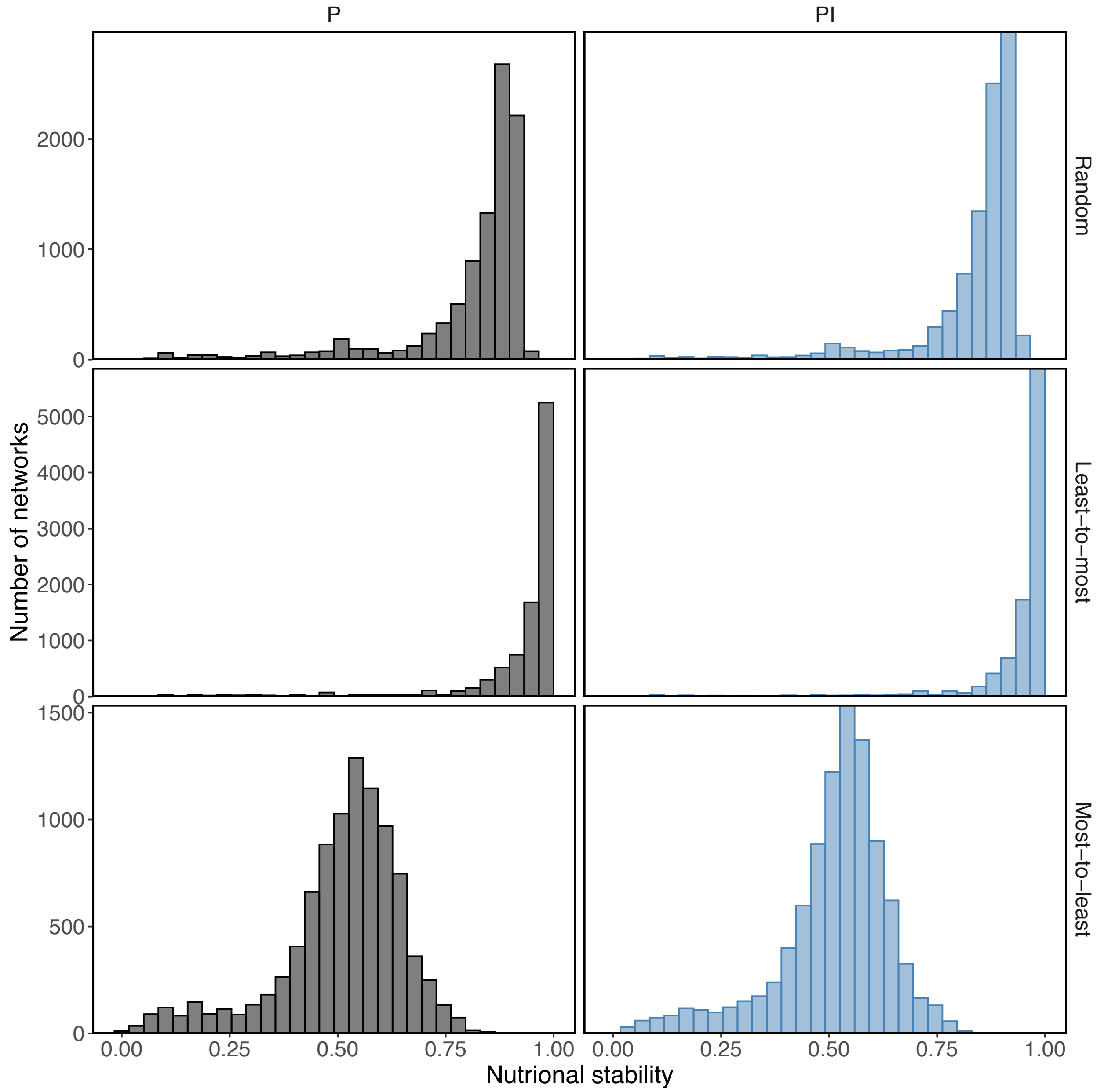

Supplementary Figure 5. Nutritional stability values of networks based on different crop removal order. Throughout the main manuscript we present nutritional stability (R_N) of networks derived from permutation of randomized crop removal order (1st row). However, removal order can also be directed. Removing crops in order of least-to-most connected (i.e. from those containing the fewest nutrients to those containing the most) generated larger R_N values (2nd row), whereas removing crops from most-to-least connected reduced R_N values (3rd row).

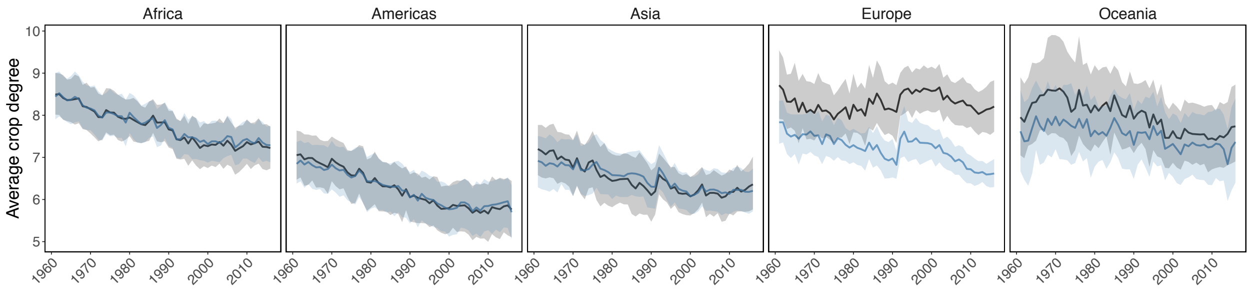

Supplementary Figure 6. Average degree of crops in crop-nutrient networks decreased over time. Only Europe exhibited source-dependent differences, with production plus imports (blue) decreasing more than production alone (black), see Supplementary Table 4 for statistics. Trend lines depict means ± 95% confidence intervals.
