# Supplementary material for: Global relationships between crop diversity and nutritional stability
Source: Nat Commun. 2021 Sep 7;12:5310. doi: 10.1038/s41467-021-25615-2 (PMC8423801; doi:10.1038/s41467-021-25615-2)
Supplement: Supplementary file 3 — Reporting Summary [file 41467_2021_25615_MOESM3_ESM.pdf]

nature research

Corresponding author(s): Charlie C Nicholson

Last updated by author(s): Jul 29, 2021

Reporting Summary

Nature Research wishes to improve the reproducibility of the work that we publish. This form provides structure for consistency and transparency in reporting. For further information on Nature Research policies, see our Editorial Policies and the Editorial Policy Checklist.

Statistics

For all statistical analyses, confirm that the following items are present in the figure legend, table legend, main text, or Methods section.

n/a	Confirmed	
☐	☒	The exact sample size (*n*) for each experimental group/condition, given as a discrete number and unit of measurement
☐	☒	A statement on whether measurements were taken from distinct samples or whether the same sample was measured repeatedly
☐	☒	The statistical test(s) used AND whether they are one- or two-sided<br>*Only common tests should be described solely by name; describe more complex techniques in the Methods section.*
☐	☒	A description of all covariates tested
☐	☒	A description of any assumptions or corrections, such as tests of normality and adjustment for multiple comparisons
☐	☒	A full description of the statistical parameters including central tendency (e.g. means) or other basic estimates (e.g. regression coefficient) AND variation (e.g. standard deviation) or associated estimates of uncertainty (e.g. confidence intervals)
☐	☒	For null hypothesis testing, the test statistic (e.g. *F*, *t*, *r*) with confidence intervals, effect sizes, degrees of freedom and *P* value noted<br>*Give P values as exact values whenever suitable.*
☒	☐	For Bayesian analysis, information on the choice of priors and Markov chain Monte Carlo settings
☐	☒	For hierarchical and complex designs, identification of the appropriate level for tests and full reporting of outcomes
☒	☐	Estimates of effect sizes (e.g. Cohen's *d*, Pearson's *r*), indicating how they were calculated

Our web collection on statistics for biologists contains articles on many of the points above.

Software and code

Policy information about availability of computer code

Data collection	No new data was collected for this work. No software was used in data collection.
Data analysis	The algorithm used to calculate nutritional stability was built in Python v 3.7.4. All statistical analyses were performed in R statistical software v 3.6.1 using the packages nlme v. 3.1-143 and lme4 v. 1.1-18-1

For manuscripts utilizing custom algorithms or software that are central to the research but not yet described in published literature, software must be made available to editors and reviewers. We strongly encourage code deposition in a community repository (e.g. GitHub). See the Nature Research guidelines for submitting code & software for further information.

Data

Policy information about availability of data

All manuscripts must include a data availability statement. This statement should provide the following information, where applicable:

- Accession codes, unique identifiers, or web links for publicly available datasets
- A list of figures that have associated raw data
- A description of any restrictions on data availability

All data used in this manuscript is publicly available. The crop production quantity data used in this study are available from the FAO Stat database [http://www.fao.org/faostat/en/#data/QCL]. The GENuS nutrient composition data used in this study are available from the Harvard Dataverse [https://dataverse.harvard.edu/dataverse/GENuS]. The intake data used in this study are available from the USDA Branded Food Products Database [https://data.nal.usda.gov/dataset/usda-branded-food-products-database].

Field-specific reporting

Please select the one below that is the best fit for your research. If you are not sure, read the appropriate sections before making your selection.

☐ Life sciences ☐ Behavioural & social sciences ☒ Ecological, evolutionary & environmental sciences

For a reference copy of the document with all sections, see nature.com/documents/nr-reporting-summary-flat.pdf

Ecological, evolutionary & environmental sciences study design

All studies must disclose on these points even when the disclosure is negative.

Study description	Using 55 years of food production data across 184 countries, we assemble over 19,000 bipartite crop-nutrient networks. We then quantify network tolerance simulated via sequential crop loss to quantify nutritional stability and crop diversity across countries, over time and between crop supply scenarios (imports versus in country production)
Research sample	This study utilized existing datasets. Crop composition information came from annual national-level food production data for years 1961 to 2016 obtained from the FAOSTAT database (Food and Agriculture Organization of the United Nations; http://fao-stat.fao.org/) for 201 nations that comprise >95% of the global population. We evaluated both the production (domestically produced crops) and production plus imports for this national yield information. Crop-specific nutrient data were obtained from the Global Expanded Nutrient Supply (GENuS) database (Smith et al 2016), a global dataset of nutrient supply of reports the amount of nutrient available per 100g of edible food for 23 nutrients across 225 food categories. We focused on 17 nutrients (calcium, carbohydrates, copper, fiber, folate, iron, magnesium, niacin, phosphorous, potassium, protein, riboflavin, thiamin, vitamin A, vitamin B6, vitamin C, and Zinc), omitting calories, fats, water, ash and refuse. Linking these two datasets results in 22,400 crop-nutrient networks, one for each country, year, and food supply scenario.
Sampling strategy	We sourced crop production quantity available for all countries for all years (1961-2016) available in the FAOSTAT database. Our aim was to use all available data, however some countries were excluded from analyses (see below). Ultimately, this yielded 184 countries with 19,044 country-year networks.
Data collection	We did not collect experimental data. All data was downloaded from secure, publicly available repositories by Benjamin F. Emery in the 2019 spring
Timing and spatial scale	We did not collect experimental data. We used FAO data from 1961 to 2016 provided at the country-level
Data exclusions	Data availability varied among global regions and missing data values were more common in the early years of the data set, we therefore removed countries that did not have at least 20 years reported (N = 17 countries), leaving 184 countries and 19,044 networks.
Reproducibility	We did not perform experiments. The data we utilized is publicly available. The algorithm used to determine nutritional stability is publicly available at our GitHub repository: https://github.com/dbemerydt/nutriStability/tree/V1.0.1
Randomization	n/a: This is an analysis of publicly available data
Blinding	n/a: Blinding is not relevant for our study as we did not collect experimental data. No groups or controls were defined, nor were test performed on subjects

Did the study involve field work? ☐ Yes ☒ No

Reporting for specific materials, systems and methods

We require information from authors about some types of materials, experimental systems and methods used in many studies. Here, indicate whether each material, system or method listed is relevant to your study. If you are not sure if a list item applies to your research, read the appropriate section before selecting a response.

Materials & experimental systems

n/a	Involved in the study	
☒	☐	Antibodies
☒	☐	Eukaryotic cell lines
☒	☐	Palaeontology and archaeology
☒	☐	Animals and other organisms
☒	☐	Human research participants
☒	☐	Clinical data
☒	☐	Dual use research of concern

Methods

n/a	Involved in the study	
☒	☐	ChIP-seq
☒	☐	Flow cytometry
☒	☐	MRI-based neuroimaging
