# Supplementary material for: Global relationships between crop diversity and nutritional stability
Source: Nat Commun. 2021 Sep 7;12:5310. doi: 10.1038/s41467-021-25615-2 (PMC8423801; doi:10.1038/s41467-021-25615-2)
Supplement: Supplementary file 2 — Peer Review File [file 41467_2021_25615_MOESM2_ESM.pdf]

nature portfolio

Peer Review File

Global relationships between crop diversity and nutritional stability

Open Access This file is licensed under a Creative Commons Attribution 4.0 International License, which permits use, sharing, adaptation, distribution and reproduction in any medium or format, as long as you give appropriate credit to the original author(s) and the source, provide a link to the Creative Commons license, and indicate if changes were made. In the cases where the authors are anonymous, such as is the case for the reports of anonymous peer reviewers, author attribution should be to 'Anonymous Referee' followed by a clear attribution to the source work. The images or other third party material in this file are included in the article's Creative Commons license, unless indicated otherwise in a credit line to the material. If material is not included in the article's Creative Commons license and your intended use is not permitted by statutory regulation or exceeds the permitted use, you will need to obtain permission directly from the copyright holder. To view a copy of this license, visit http://creativecommons.org/licenses/by/4.0/.

Reviewers' Comments:

Reviewer #1:
Remarks to the Author:
Thank you for the opportunity to review this very important paper. There is a significant gap between what is grown in countries and dietary diversity and even further, nutritional outcomes. The literature shows that often, proximity to markets matter more than production.

Introduction:

The authors highlight the idea that crop diversity will not necessarily equate to nutritional outcomes, or even improved dietary diversity. That is true and perhaps should be expanded upon by the authors. People don't eat crops or commodities. They eat foods that have been manipulated, moved, and processed into complex, mixed foods. An increasing proportion of the diet is now processed packaged foods. Very few people "live off the land" outside deeply rural areas in low-income settings and some high-income farms. Even then, farmers are net buyers. The point being, crop diversity is the starting point towards ensuring more foods are available that would contribute to dietary diversity but it is not nearly sufficient enough. It would be great for the authors to acknowledge this in the introduction but also, as a limitation of their study.

The authors are using terms such as nutrient adequacy, nutritional stability, nutritional functional groups, etc that I think most readers will not understand. There is even debate about these terms in the very technical nutrition community. Perhaps some definitions of these terms up front is essential for the readers to understand what this paper is about.

Methods:

I am less able to review the methods as I am not an expert on network topology. Thus, I am sorry if some of my comments below are a bit naive. Take them as areas that could be clarified for the reader who also may not be an expert.

On the 17 nutrients chosen, why those? Some are not so important from a human nutrition standpoint. Phosphorus for example is ubiquitous. Copper is a minor mineral and copper deficiency is not a prominent issue. Vitamin D is a significant issue and is missing as are Iodine and B12. Why were these particular nutrients chosen?

I am really unclear about what is nutritional stability. Is it that the nutritional value is compromised when there is a disturbance, loss, or shock to a crop? The authors never define it. I am not sure if this is a new indicator/variable or something from the literature. I believe the former?

Figure 1:
I am sorry, but I am not completely understanding Figure 1. For A, what are the boxes with the different colors? I see that with B and C, there is a loss, and nutrients decline? But what is the difference between B and C? D and E are more clear, but I am not sure how they relate to A, B, and C. How that conclusion is made between the Maldives and China.

Overall, I believe it says that if you have more nutrient risk crops, in the face of a shock or loss, there is fewer crop loss? What does a high average crop degree mean?

Figure 2:

Is there a difference between the regions? Meaning, do some regions have less unique crops that provide more nutritional stability? You may need to more explicitly spell this out to the reader.

Figure 3A:

This is interesting because I think this is very different than the findings of Khoury et al 2014 PNAS that in most regions of the world, agriculture has become more homogenized I believe. Why do

you think you are seeing differences as compared to this analysis? Please do correct me if this is incorrect or not comparable. Is it more because trade has increased? It would be great to show some country examples from these regions to show production vs trade - and how they contributed to the diversity. This is somewhat similar to Remans et al 2014 GFS. Europe is fascinating. Would be interesting to understand this a bit more.

Khoury, C.K., Bjorkman, A.D., Dempewolf, H., Ramirez-Villegas, J., Guarino, L., Jarvis, A., Rieseberg, L.H. and Struik, P.C., 2014. Increasing homogeneity in global food supplies and the implications for food security. Proceedings of the National Academy of Sciences, 111(11), pp.4001-4006.

Remans, R., Wood, S.A., Saha, N., Anderman, T.L. and DeFries, R.S., 2014. Measuring nutritional diversity of national food supplies. Global Food Security, 3(3-4), pp.174-182.

Figure 3C:

Are the authors saying that when looking at the nutritional stability in a region, one has to look at BOTH production and trade? This is of course makes sense, but it would be good to articulate that very clearly. The way it is written is not crystal clear.

Figure 5:

This is a key graph and results. This may be my own naivete, but I just don't understand crop degree as compared to crop diversity. Perhaps the authors can explain that better earlier on or in the methods. The authors say: "Stated succinctly, even though crop diversity has increased, there are diminishing returns on nutrient availability from adding more crops into a network, especially since the crops being added into networks appear to provide fewer links to nutrients not already in the food system." This is not very clear to me.

Some questions:
- I am finding this idea of "networks" hard to relate to. Do the authors mean landscapes? And if landscapes, regional landscapes?
- What do you mean by "provide fewer links to nutrients"?

Discussion:
You are not linking to nutritional outcomes. Nutritional outcomes are indicators such as stunting, wasting, BMI. You are linking to nutrients that are constituents of food that then makeup diets. I would be clear on the outcomes you are measuring.

Overall, I find the discussion very thoughtful and helps explain the paper a bit more along with the limitations of the results.

Reviewer #2:
Remarks to the Author:
Review of "Global relationships between crop diversity and nutritional stability" by Nicholson et al.

The authors assembled bipartite networks of food crops connected to nutrients and measured "nutritional stability" by simulating how the loss of crops would change nutrient provision. They find a positive, saturating relationship between crop diversity and nutritional stability in multiple countries. They also studied changes in nutritional stability through time and the role of imported crops.

The data set is cool and I like the general approach of using network node-removal models ("secondary extinction" models in ecology) to assess nutrient provision. However, I need more convincing that the current methods are comprehensive enough to justify the claims made in the manuscript. I have three major comments: (1) Include analysis of nutrient provision in addition to nutritional stability; (2) Expand network analysis; and (3) Discuss network analysis limitations. I

provide details on these issues below (along with some minor comments), and would like to hear the authors' response before I can be supportive of publication.

(1) Include analysis of nutrient provision in addition to nutritional stability

Nutritional stability is an interesting concept, but it is first important to establish how much variation there is between countries and between time points in terms of nutrient provision (#nutrients in a network; as per the data set). In terms of interpreting nutritional stability, it is clearly relevant to know if high/low nutritional stability (a measure that does not directly account for either nutrient provision or crop diversity) is associated with a country that has high/low starting nutrient provision. In addition, a plot of nutrient provision against crop diversity would be informative (similar to Fig. 2).

(2) Expand network analysis

The current network analysis is pretty bare-bones. One example is that only a single node-removal scenario is considered: right now, crops are removed sequentially at random. The general practice has been to include at least two additional removal scenarios: removing nodes from most-to-least connected and from least-to-most connected. Doing so would provide some idea of a lower and upper bound, respectively, to nutritional stability for a given network. Even better is to also consider more realistic removal scenarios, in this case, perhaps based on trade patterns or climate change risk to food supply.

Indeed, the current approach could be considered somewhat out-of-date given the emergence of a Bayesian network approach to analyzing secondary extinctions in networks (Eklöf et al. 2013). (And see Staniczenko et al. 2017 for an introduction to Bayesian networks more generally in ecology.) One of the main advantages of the Bayesian network approach is that it does not require multiple simulation runs for each network, and provides a robustness (here, nutritional stability) measure that accounts for all possible runs in a much shorter amount of time (because it does so analytically). It also allows different functional forms to be specified for how the loss of crops would affect nutrient stability (rather than a nutrient is lost only when all crops that provide that nutrient are lost).

In any case, in addition to considering additional removal scenarios, motivated by the issue I outline in Major Comment 1 it would be worth repeating the analysis with a slightly different measure of nutritional stability. Currently, the maximum number of nutrients is determined on a network-by-network basis (see Fig. 1). However, this approach does not account for different baseline nutrient provision in different countries and time periods. As such, it would be interesting to repeat the analysis but with a network-independent maximum for the number of nutrients (i.e., nutrient provision) on the y-axis. Nutritional stability could then be given in terms of the area realized compared to the maximum possible area achievable (i.e., consistent with maximum number of nutrients remaining until essentially all crops are removed from a network).

In fact, this additional analysis motivates a number of questions that, surprisingly, are not mentioned in the manuscript: What is the optimum crop portfolio for a country to hold? What is the similarity in crop portfolios (and nutrient availability) among countries?

My main point is that nutritional stability on its own may not tell the whole story.

(3) Discuss network analysis limitations

The manuscript includes much discussion (P8 to P11) of the implications of their main finding (that nutritional stability increases then saturates with increasing crop diversity) but less about the limitations of their approach, which should be addressed.

The effects of not including relative crop abundances should be discussed. Right now, the probability of losing a crop is the same for all crops, but this is hardly realistic. How much would results be affected if, for example, less abundant crops were lost first? It would also be better to consider the effect of a fractional loss of crops on nutrient provision, rather than the current, "all

or nothing" approach. It is worth noting that these considerations---probabilistic loss and fractional loss---are implemented in the Bayesian network approach (Eklöf et al. 2013).

Network rewiring (e.g., Staniczenko et al. 2010; Vizentin-Bugoni et al. 2020) should also be discussed, both in terms of previous work and next steps. While rewiring in the crop-nutrient networks would be different from in ecological networks (a predator instigates a new trophic interaction if it loses a prey item), it is still likely to happen. For example, it is unlikely a country will do nothing as it loses all its crops, but would instead begin to import comparable crops to fill the nutritional deficit. It is worth noting that, in addition to implementations of rewiring in the authors' current approach (see Staniczenko et al. 2010 and Vizentin-Bugoni et al. 2020 for examples), a method for rewiring has been proposed in the Bayesian network approach (Baldock et al. 2019).

REFERENCES

Baldock, K.C.R. et al. (2019). A systems approach reveals urban pollinator hotspots and conservation opportunities. Nat. Ecol. Evol., 3, 363–373.

Eklöf, A., Tang, S. & Allesina, S. (2013). Secondary extinctions in food webs: a Bayesian network approach. Methods Ecol. Evol., 4, 760--770.

Staniczenko, P.P.A., Lewis, O.T., Jones, N.S. & Reed-Tsochas, F. (2010). Structural dynamics and robustness of food webs. Ecol. Lett., 13, 891--899.

Staniczenko, P.P.A., Sivasubramaniam, P., Suttle, K.B. & Pearson, R.G. (2017). Linking macroecology and community ecology: Refining predictions of species distributions using biotic interaction networks. Ecol. Lett., 20, 693–707.

Vizentin-Bugoni et al. (2020). Including rewiring in the estimation of the robustness of mutualistic networks. Methods Ecol. Evol., 11, 106--116.

MINOR COMMENTS

-- Include in the main text a brief description of the methods workflow and an interpretation of R_N.

-- P7. Sentence beginning, "Stated succinctly..." It is worth noting that adding low-degree crops may still be worthwhile if they are linked to "vulnerable" nutrients that may only be provided by one or two existing crops (and see comment directly below).

-- P7. "...stability depends on the number of link within this network." Stability also depends on the *pattern* of connections in the network (otherwise, there would be no need to perform a network analysis and node removal simulations).

-- P12. How many crops are considered in total? 225?

-- P14. How many runs were conducted for each network before averaging?

-- P14. How was the "average attack tolerance" calculated exactly? (Note that this terminology is unconventional: "attack" is typically used to reference removing high-degree nodes first, whereas "error" is typically used to reference random removal.)

-- P15. Give more details on the autoregressive model. How does it connect to the R_N variance shown in Fig 3B?

-- P22. Consider including in Fig. 2 separate panels associated with different time periods, e.g., 1990 to 2000.

-- P23. How are the continental values in Panel 3 calculated?

-- P25. What is the numerical relationship for the linear regression?

Reviewer #3:
None

NB: Please see our track changes document for reference to line numbers

Reviewer #1 (Remarks to the Author):

Thank you for the opportunity to review this very important paper. There is a significant gap between what is grown in countries and dietary diversity and even further, nutritional outcomes. The literature shows that often, proximity to markets matter more than production.

> Thank you for taking the time to review our manuscript. We agree, dietary diversity is a complicated, but necessary, field of study. We appreciate your input and calls for us to be more clear around how we define and describe nutritional stability.

Introduction*:*

The authors highlight the idea that crop diversity will not necessarily equate to nutritional outcomes, or even improved dietary diversity. That is true and perhaps should be expanded upon by the authors. People don't eat crops or commodities. They eat foods that have been manipulated, moved, and processed into complex, mixed foods. An increasing proportion of the diet is now processed packaged foods. Very few people "live off the land" outside deeply rural areas in low-income settings and some high-income farms. Even then, farmers are net buyers. The point being, crop diversity is the starting point towards ensuring more foods are available that would contribute to dietary diversity but it is not nearly sufficient enough. It would be great for the authors to acknowledge this in the introduction but also, as a limitation of their study.

> You raise an important point, which is that crop diversity is a starting point for understanding food/nutrient availability. We address this issue, in part, when discussing how our work fits in within the classical pillars of food systems (L260-268). However, we agree that this issue should be raised up front, so we have added the following text to the Introduction (L45-47):
>
> "Crop diversity is a starting point towards ensuring more foods are available that would contribute to dietary diversity, but it is not itself a nutritional outcome."

The authors are using terms such as nutrient adequacy, nutritional stability, nutritional functional groups, etc that I think most readers will not understand. There is even debate about these terms in the very technical nutrition community. Perhaps some definitions of these terms up front is essential for the readers to understand what this paper is about.

> Thank you for bringing this up. Throughout, we want to be as clear as possible about the different aspects of nutritional ecology that we are addressing or discussing. We have tried to limit the number of terms we use (e.g. replacing

"nutritional functional groups" with "distinct nutrients"). And further define nutritional stability (L55-56, L67-68).

Methods:

I am less able to review the methods as I am not an expert on network topology. Thus, I am sorry if some of my comments below are a bit naive. Take them as areas that could be clarified for the reader who also may not be an expert.

Considering a group foods and their constituent nutrients as a biological network may not be immediately intuitive. Your suggestions are actually quite helpful in pushing us to be more clear about our approach. Reviewer 2 has an excellent grasp of the network approaches we apply. They have provided many helpful methodological suggestions.

On the 17 nutrients chosen, why those? Some are not so important from a human nutrition standpoint. Phosphorus for example is ubiquitous. Copper is a minor mineral and copper deficiency is not a prominent issue. Vitamin D is a significant issue and is missing as are Iodine and B12. Why were these particular nutrients chosen?

We were limited to the nutrients analyzed in the GENUS dataset: calories, fat, protein, carbohydrates, dietary fiber, vitamin C, vitamin A, folate, thiamin, riboflavin, niacin, total B6, calcium, iron, zinc, potassium, copper, magnesium, selenium, phosphorus, saturated fatty acids, monounsaturated fatty acids, and polyunsaturated fatty acids. The 17 nutrients we focused on were chosen because we wanted to focus on crop-derived micro-nutrients, and so we excluded calories, dietary fiber, and the various fats (L324-325). We have noted this reason in the methods, including a recognition that we do not include some important globally deficient micronutrients including Vitamin D, Iodine, and B12 (L322-327).

I am really unclear about what is nutritional stability. Is it that the nutritional value is compromised when there is a disturbance, loss, or shock to a crop? The authors never define it. I am not sure if this is a new indicator/variable or something from the literature. I believe the former?

Apologies that this is not more clear. One thought experiment that we have found helpful is to consider two baskets at the grocery store. In one basket, there are three sources of Vitamin C: mangoes, oranges and papaya. A second basket just has papaya. If papaya is removed from both baskets, then the first still has available sources of vitamin C (mangoes & oranges). However, the second basket can no longer offer any vitamin C. Within the context of this simple example, we would say that the first basket has greater nutritional stability than the second. Because the first basket has redundant sources of vitamin C it is has a greater capacity to provide nutrients even when food items are removed.

We define nutritional stability (L55-56) as "the capacity of a food system to provide sufficient nutrients despite disturbance". We are providing a new systems-level indicator that captures how resilient food systems are to loss of their components, in this case crops. To make this new indicator more clear we have stressed what this metric does/does not measure (L372-375):

"This unitless measure represents how robust a food system is to the sequential elimination of crops. It does not provide information on the identity of different crops or nutrients, nor the total availability of nutrients, the utilization of nutrients or the nutrient adequacy of a selection of foods.

Figure 1:
I am sorry, but I am not completely understanding Figure 1. For A, what are the boxes with the different colors?

The boxes with different colors represent hypothetical crops. These different crops possess different sets of nutrients (circles). We consider these crops and their nutrients a bipartite (2-level) ecological network.

I see that with B and C, there is a loss, and nutrients decline? But what is the difference between B and C? D and E are more clear, but I am not sure how they relate to A, B, and C. How that conclusion is made between the Maldives and China.

B & C show that when we remove crops from a network, the number of nutrients remaining in the network decreases, *but the order that you remove these crops determines the number of nutrients remaining*. For example, if the 1st crop lost is the "blue crop" (left example below), then the blue line in B decreases from 4 to 2 remaining nutrients because this crop possesses 2 nutrients (the 3rd and 4th circles) that are not found in any other crop. Contrast this to the "yellow crop" which possesses a single nutrient and this nutrient is shared with the "orange crop" and "red crop". Because of this redundancy, if the 1st crop removed is the "yellow crop" (right example below) no nutrients are immediately lost, as is shown in C.

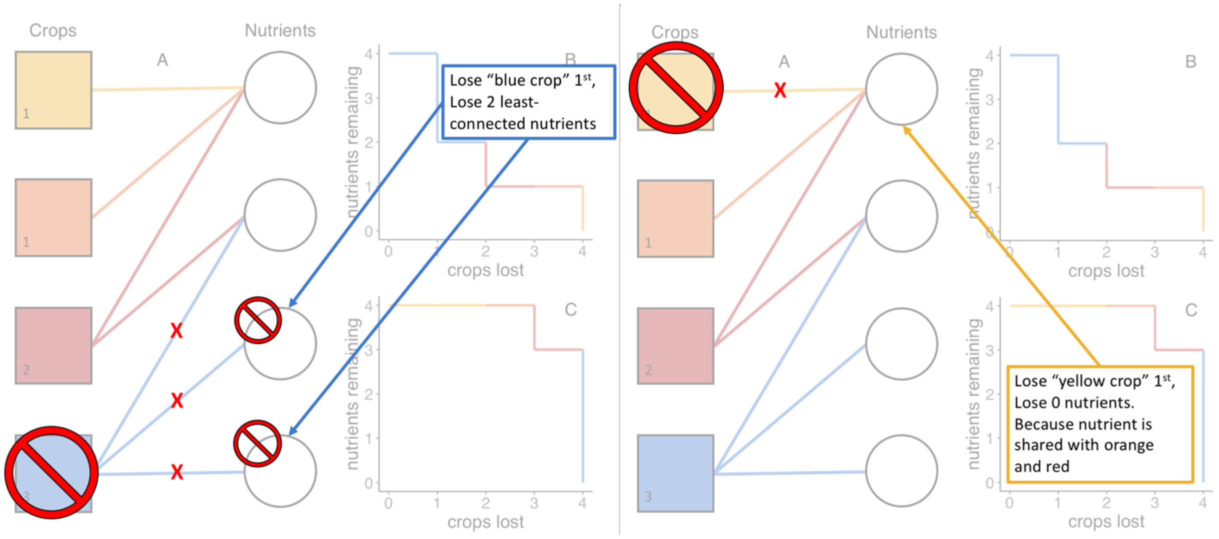

A common method in network science to account for these priority effects is to randomize removal order many times and create an average removal curve. In D and E we are showing actual examples of multiple randomized removal sequences (light gray lines) and the average removal curve (black line).

We have updated the caption of Figure 1 and we hope that it helps clarify this and other issues.

Moreover, prompted by comments from Reviewer 2, we have included in this revised manuscript the distribution and trends of nutritional stability when we considered two additional removal scenarios: removing the most-connected crops first (most-to-least removal) and removing least-connected first (vise versa). If you are curious, please see Figures S4 and S5, and discussion of these below.

Overall, I believe it says that if you have more nutrient risk crops, in the face of a shock or loss, there is fewer crop loss?

This interpretation is very close to our conclusion. If you have many redundant nutritional links (i.e., the same nutrient is provided by multiple crops (e.g., nutrient #1 in A)), in the face of a shock or loss you will be less likely to lose nutrients.

What does a high average crop degree mean?

Degree is a measure in network science that is the number of connections a given "node" has. In this case, it's the number of nutritional links that a given crop has. Each crop possesses a unique number of nutrients. For example, in Figure 1 the "blue crop" possesses 3 nutrients, therefore it has a degree of 3. We can also calculate the average degree of the whole network. In the case of the 4

crops shown in Figure 1, the average crop degree is 3.5 ((1 + 1 + 2 + 3)/4). We have tried to further clarify this in the intro (L71-75):

"Importantly, crop species identity can affect overall network structure; a nutrient rich crop will have greater degree (i.e. more connections within the network; Fig. 1A). A network with many nutrient-rich crops will possess high average crop degree (i.e. many crops that all have many nutrients), and is thus more robust to having individual crops lost from the network, because other crops likely contain the same nutrients."

And have added the following text to the caption of Figure 1 to explain degree more clearly:

"Species identity matters; crops with more nutritional links (i.e. node degree; values within boxes) contribute more to overall stability. Networks with higher average degree (total links/total crops) are more robust to crop removal."

Figure 2:

Is there a difference between the regions? Meaning, do some regions have less unique crops that provide more nutritional stability? You may need to more explicitly spell this out to the reader.

Thanks for pushing us to do so. Based on this comment and those by Reviewer 2 we have added a supplemental figure (Figure S2) that compares crop diversity between regions for both production and production + imports (see histograms below). We report this in our results (L107-111):

"The average number of crops in a given country's network varies between regions (range: 12-27), yet 83% of all networks possessed all 17 nutrients we examined here (Fig. S2). We also found positive, non-linear relationships between crop diversity and nutrient diversity (i.e. more crops is associated with more nutrients available), and a linear relationship between nutrient diversity and nutritional stability (Fig. S3)."

Figure 3A:

This is interesting because I think this is very different than the findings of Khoury et al 2014 PNAS that in most regions of the world, agriculture has become more homogenized I believe. Why do you think you are seeing differences as compared to this analysis? Please do correct me if this is incorrect or not comparable. Is it more because trade has increased? It would be great to show some country examples from these regions to show production vs trade - and how they contributed to the diversity. This is somewhat similar to Remans et al 2014 GFS. Europe is fascinating. Would be

interesting to understand this a bit more.

Thank you for raising this point. Khoury et al.'s paper was foundational to the present work because they applied concepts from community ecology to food systems. Khoury et al. showed that cropping systems have homogenized. In regards to crops, we depict something different, but actually more simple. We're depicting how crop diversity has changed, whereas Khoury showed how crop composition has homogenized.

To return to the grocery basket example, you and I could both have 4 items (crop diversity = 4), but the identity of these items is completely different (tomatoes, peppers, potatoes, & millet *vs.* mangoes, oranges, papaya & carrots). Fast forward 2 weeks, you and I both have 6 items in our basket (crop diversity = 6) and they are exactly the same (tomatoes, peppers, potatoes, millet, mangoes, & oranges). *You can increase the number of different items present, and yet they can become more similar*. Khoury et al. performed a similarity analysis (via PERMANOVA) to show how composition changed, we depict how the diversity of crops has changed. In other words, our analysis is concerned about the outcome of 6- the diversity of the basket itself. Khoury is concerned with the fact that after two weeks, both of our baskets are the **same** 6 items- i.e. we have completely the same crops.

Remans et al. work has also been very informative. While we for the most part do not get into country level comparisons in the present work, we believe that the other issues you raised ("*production vs trade - and how they contributed to the diversity")* are illustrated in Fig 4, where we see strong differences between production and production + imports scenarios in terms of nutritional stability. We have also produced similar results and figures for crop diversity which we present in the supplemental materials (Fig S2) and report in the Results L107-111.

***Figure 3C**:*

Are the authors saying that when looking at the nutritional stability in a region, one has to look at BOTH production and trade? This is of course makes sense, but it would be good to articulate that very clearly. The way it is written is not crystal clear.

Thank you for pushing us to be more clear on this point. Based on your suggestions, we have added the following to the Results text (L153-154):

"When looking at the nutritional stability of a region, it is therefore important to consider both production and trade."

***Figure 5**:*

This is a key graph and results. This may be my own naivete, but I just don't understand crop degree as compared to crop diversity. Perhaps the authors can explain that better earlier on or in the methods. The authors say: "Stated succinctly, even though crop diversity has increased, there are diminishing returns on nutrient availability from adding more crops into a network, especially since the crops being added into networks appear to provide fewer links to nutrients not already in the food system." This is not very clear to me.

We agree that this is an important finding of this work, so we want to make sure that is clear. As per your comments above we have added more description of crop degree in both the introduction (L71-75), and Figure 1 caption, and we expand on this in the methods (L343-346). For an individual crop, its degree is simply how many nutrients it possesses (i.e., the number of "nutritional links" it has). For multiple crops, average crop degree (as is shown in Fig.5) is the average number of nutritients across all crop in a food system (i.e. total number of links/total number of crops per network). High average crop degree in a food system is indicative of greater redundancy and therefore stability. Conversely, crop diversity is more simply the number of crops in a food system. Hopefully our description of Fig. 1 above also makes this more clear.

Some questions:
- I am finding this idea of "networks" hard to relate to. Do the authors mean landscapes?

We use network science as a way to link crops to nutrients, which is part of the novelty of this work. At the same time we recognize that networks can be an abstract concept for some. We hope that the changes that we have made throughout the MS and our comments herein help clarify this unique approach to exploring food systems.

And if landscapes, regional landscapes?

We do not mean landscapes. However the method that we describe here can be applied to a collection of foods obtained at any scale, from the grocery basket to household intake to national food balance data, as is done here.

- What do you mean by "provide fewer links to nutrients"?

We mean that these crops possess fewer different types of nutrients, and therefore provide fewer connections within the crop-nutrient network and contribute less to overall stability.

Discussion:
You are not linking to nutritional outcomes. Nutritional outcomes are indicators such as stunting, wasting, BMI. You are linking to nutrients that are constituents of food that then makeup diets. I would be clear on the outcomes you are measuring.

Very true, thank you for highlighting this. We are more careful now and state what we actually report, changes in a nutritional measure. We have removed use of the term ‘nutritional outcomes’ throughout. We think that correlating our nutritional stability metric to health outcomes, such as those you identify, could be very interesting but is beyond the scope of the present work. In addition to our caveats (L261-268), we have added language to make your point clear, stating (L208-213):

“To be clear, we are measuring the relationships of crop diversity to nutrients and their susceptibility to disturbance; we are not measuring nutritional outcomes such as dietary intake, dietary diversity, or other health-related outcomes that are the result of nutrition. Just as nutritional status cannot be determined from dietary intake alone, nutritional stability does not determine the availability, let alone utilization, of nutrients. This is however, a natural area to expand this work moving forward.”

Overall, I find the discussion very thoughtful and helps explain the paper a bit more along with the limitations of the results.

Thank you. We hope that the revisions we have made and some of our explanatory comments make the manuscript more clear and compelling. Thank you for your thoughtful comments that have pushed us to be more clear. And thank you for taking the time to review this manuscript.

Reviewer #2 (Remarks to the Author):

Review of "Global relationships between crop diversity and nutritional stability" by Nicholson et al.

The authors assembled bipartite networks of food crops connected to nutrients and measured "nutritional stability" by simulating how the loss of crops would change nutrient provision. They find a positive, saturating relationship between crop diversity and nutritional stability in multiple countries. They also studied changes in nutritional stability through time and the role of imported crops.

The data set is cool and I like the general approach of using network node-removal models ("secondary extinction" models in ecology) to assess nutrient provision. However, I need more convincing that the current methods are comprehensive enough to justify the claims made in the manuscript. I have three major comments: (1) Include analysis of nutrient provision in addition to nutritional stability; (2) Expand network analysis; and (3) Discuss network analysis limitations. I provide details on these issues below (along with some minor comments), and would like to hear the authors' response before I can be supportive of publication.

> Thank you for taking the time to review this manuscript. We are pleased that you found the dataset and approach cool. We strive to address these three major comments below. In general, we aimed to balance the calls from reviewer 1 for clarity with your astute methodological suggestions and calls for expanding on certain aspects. This is sometimes a tight line to walk, but we do our best.

(1) Include analysis of nutrient provision in addition to nutritional stability

Nutritional stability is an interesting concept, but it is first important to establish how much variation there is between countries and between time points in terms of nutrient provision (#nutrients in a network; as per the data set). In terms of interpreting nutritional stability, it is clearly relevant to know if high/low nutritional stability (a measure that does not directly account for either nutrient provision or crop diversity) is associated with a country that has high/low starting nutrient provision. In addition, a plot of nutrient provision against crop diversity would be informative (similar to Fig. 2).

> This is an excellent point. We agree that it would be useful to better understand the relationship between nutritional stability and # of nutrients in a network. We find that # nutrients increases with the number of crops non-linearlyand that there is a linear relationship between # of nutrients and nutrient stability (see below). While we believe that presenting this analysis is valuable, we don’t think this outcome changes the interpretation of our main results. This is because the majority (83%) of all crop-nutrient networks contain the maximum number of possible nutrients, therefore most country-years have a similar baseline in terms of “nutrient-provision”

We have added these plots to the supplemental materials (Fig. S2 & S2), as well as report these relationships in the results (L107-111). Based on comments from Reviewer 1 we are trying to limit additional nutrition terminology, such as “nutrient provision”, which may mean different things to a nutritionist vs. a food system scientist. As this measure (i.e. “*(#nutrients in a network; as per the data set”*) is analogous to our crop diversity measure, we instead report it as “nutrient diversity”.

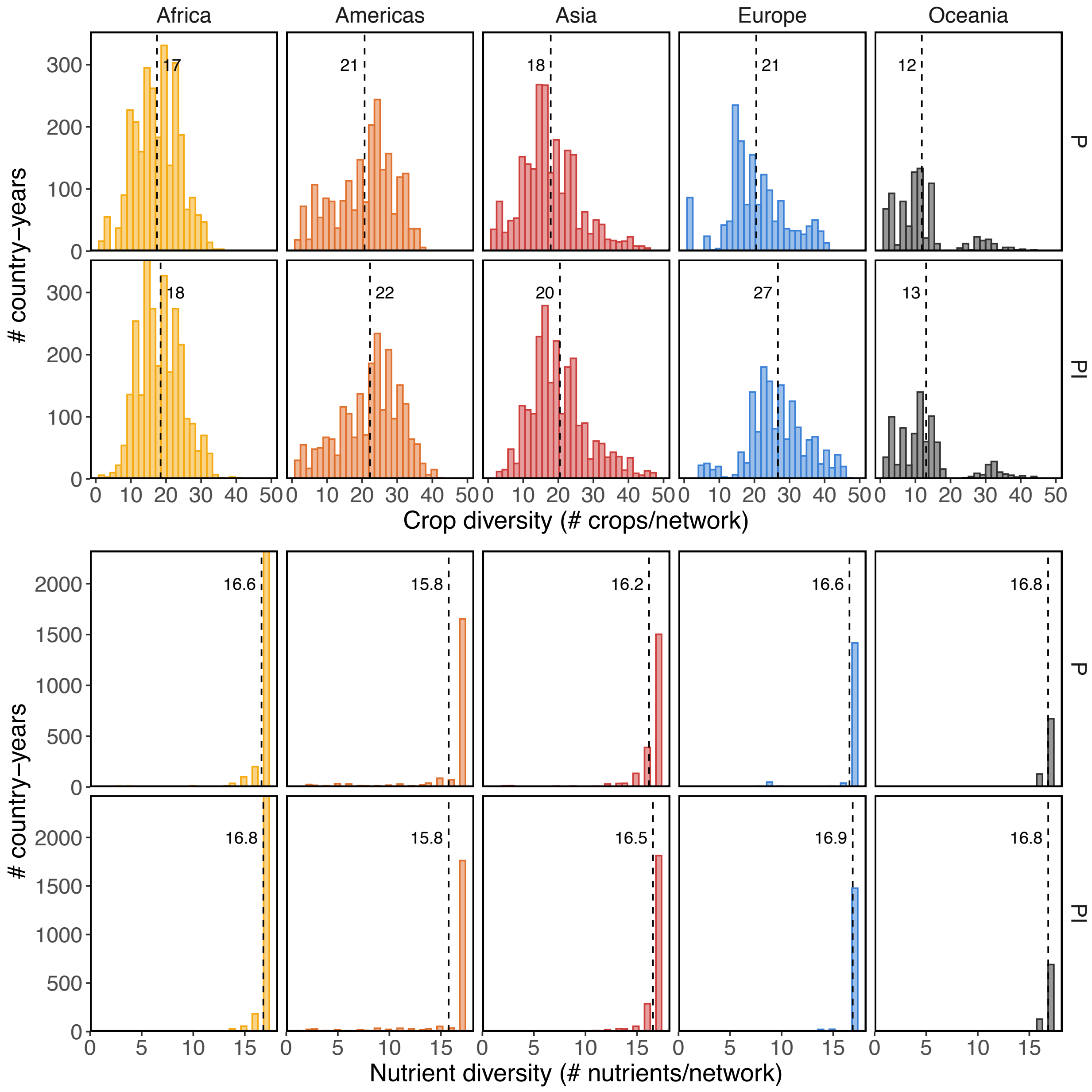

Fig. S2. Distributions of crop and nutrient diversity for regions and supply scenarios. Each bar depicts a region’s number of networks for separate country-year combinations belonging to a specific levels of crop diversity (A) or nutrient diversity (B) for both production (P; top rows) and production and imports (PI; bottom rows)

scenarios. Average values across countries and years are provided and depicted by the dashed vertical line. Crop diversity could be comprised of 225 different FAO food balance crop commodities. There are 17 micro-nutrients available in the GeNUS dataset that we analyzed. Over 83% of all crop nutrient networks (N = 19044) possessed all 17 micro-nutrients that we analyzed here.

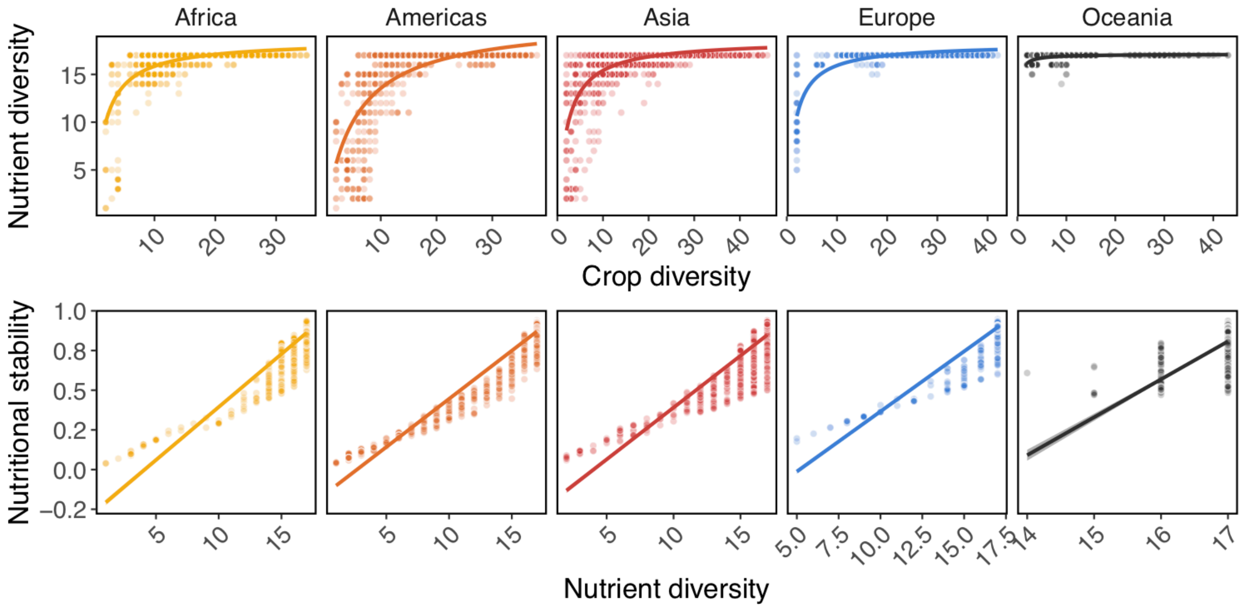

Fig. S3. Nutrient diversity increases with crop diversity and is associated with greater nutritional stability. Each point represents the crop diversity, nutrient diversity or nutritional stability from a country's crop-nutrient network in aa given year. Non-linear relationships were fitted with same saturating function (α *x/(β + x)) as in the main text.

(2) Expand network analysis

The current network analysis is pretty bare-bones. One example is that only a single node-removal scenario is considered: right now, crops are removed sequentially at random. The general practice has been to include at least two additional removal scenarios: removing nodes from most-to-least connected and from least-to-most connected. Doing so would provide some idea of a lower and upper bound, respectively, to nutritional stability for a given network. Even better is to also consider more realistic removal scenarios, in this case, perhaps based on trade patterns or climate change risk to food supply.

Thank you raising this, it is a point that we are aware of and discussed in early formulations of this research. As always, we strived to balance introducing a new, interdisciplinary approach with impactful results, while doing so succinctly and with clear language and methods. We believe that this is already an information- and result-dense manuscript as is, and we aimed to keep analyses

straightforward. However, we agree that a standard is to consider more than one removal sequence. We have added an additional figure to the supplement (similar to our Figure 3C) and histograms that depict the upper/lower bounds of nutritional stability based on least/most-connected removal order (Fig. S4, S5). We report these findings in the Results (L141-144):

"The direction of these trends did not change with different crop removal procedures (see methods; Fig. S4), however the magnitude of nutritional stability was lower when crops were removed in order of most-to-least connected (Fig. S5)."

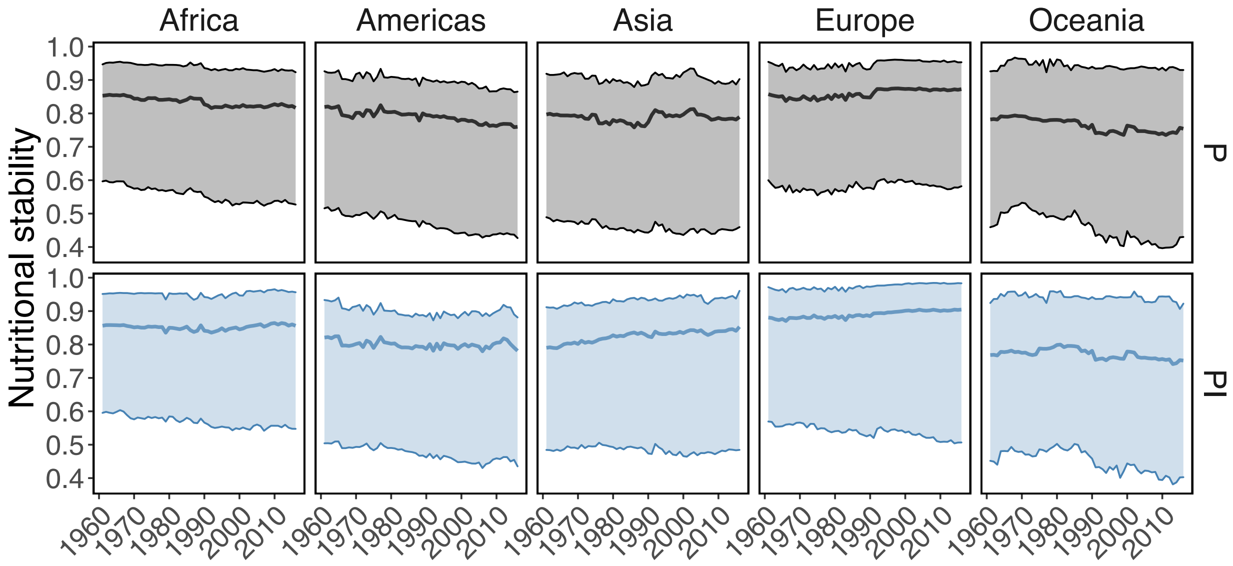

Fig. S4. Trends of nutritional stability considering different crop removal procedures. Nutritional stability (R_N) can be calculated different ways by changing the removal sequence of crops. In the main manuscript we report R_N values based on randomized crops loss. We also ordered crop loss from most to least connected crops (i.e. from those containing the most nutrients to those containing the fewest), and vice versa. Here we show trends in randomized R_N (solid middle line) with an upper bound derived from least-to-most removal and a lower bound derived from most-to-least removal for both production (P; top row) and production + imports (PI; bottom row) scenarios.

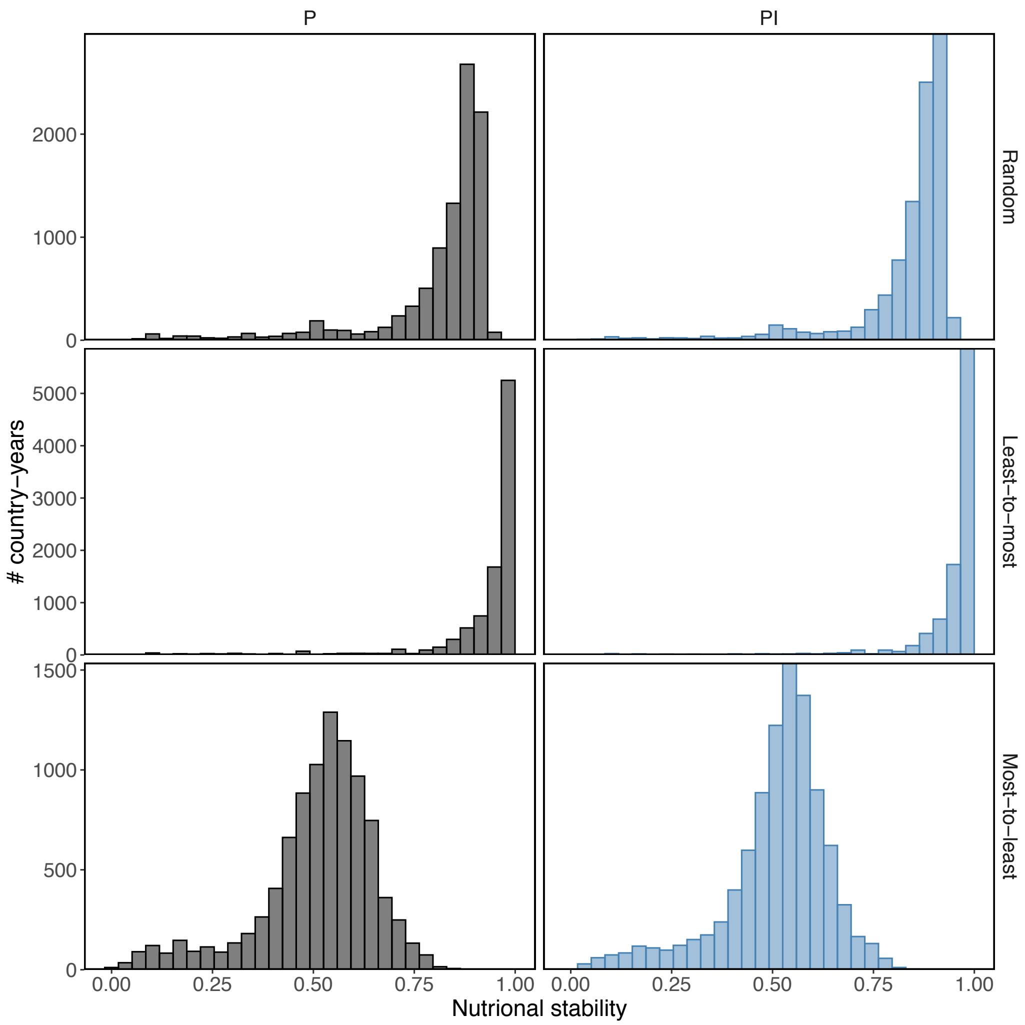

Fig. S5. Nutritional stability values of networks based on different crop removal order. Throughout the main manuscript we present nutritional stability (R_N) of networks derived from permutation of randomized crop removal order (1st row). However, removal order can also be directed. Removing crops in order of least-to-most connected (i.e. from those containing the fewest nutrients to those containing the most) generated larger R_N values (2nd row), whereas removing crops from most-to-least connected reduced R_N values (3rd row).

Indeed, the current approach could be considered somewhat out-of-date given the emergence of a Bayesian network approach to analyzing secondary extinctions in networks (Eklöf et al. 2013). (And see Staniczenko et al. 2017 for an introduction to Bayesian networks more generally in ecology.) One of the main advantages of the Bayesian network approach is that it does not require multiple simulation runs for each network, and provides a robustness (here, nutritional stability) measure that accounts for all possible runs in a much shorter amount of time (because it does so analytically). It also allows different functional forms to be specified for how the loss of crops would affect nutrient stability (rather than a nutrient is lost only when all crops that provide that

nutrient are lost).

Thank you for suggesting this approach and advising on appropriate literature. Aggregating posterior secondary extinction probabilities as a measure of robustness is indeed an up-to-date, Bayesian approach. We have looked into whether retooling our entire analytical approach using a Bayesian network approach makes sense. We offer three reasons for continuing to use our current approach:

1. One of the main advantages you highlight of a Bayesian approach is speed. As we have already calculated network stability, efficiency gains through analytical solutions are somewhat moot. However, we highlight that for future work a Bayesian network approach would yield efficiency gains, stating in the Methods (L370-372): "It is worth noting a Bayesian network approach could be used to efficiently derive a similar robustness metric by aggregating posterior secondary removal probabilities via analytical solutions, rather than permutation".

2. We find the permutation-based approach that we use is intuitive and is easily explained graphically, as we do in Figure 1. We believe that this is important given that we are putting forth a new and somewhat abstract concept: crop-nutrient networks. As Reviewer 1 highlights, there are some conceptual challenges in this paper, especially for those not immersed in network science. The conceptual complexity of Bayesian network approaches may outweigh the comprehensibility of our current approach.

3. Our understanding is that a Bayesian network approach would require user-defined parameterization at two steps.
    a. First, it requires the specification of a *prior probability* of extinction for each species in a network. It is not exactly clear to us how this is best done. In past work (e.g., Baldock et al. 2019, Häussler et al. 2020), these probabilities have been defined by total abundance or the amount of resources available to secondary consumers. In our approach we consider all species (crops) as equally likely to be removed. Although simple, we believe that this approach has merits in that it does not impose *a priori* expectations about crop loss probabilities, which are likely to be highly dynamic and regionally dependent.
    b. Second, the functional forms presented by Eklöf et al. 2013 each come with their own expectations about whether loss is (non)monotonic. While we can imagine scenarios in which extinction probability increases nonlinearly with crop loss, defining and rationalizing the slope parameters (their α and β) seems beyond the scope of the present work.

Baldock, K.C.R. et al. (2019). A systems approach reveals urban pollinator hotspots and conservation opportunities. Nat. Ecol. Evol., 3, 363–373.

Häussler, J., Barabás, G., & Eklöf, A. (2020). A Bayesian network approach to trophic metacommunities shows that habitat loss accelerates top species extinctions. *Ecology letters*, *23*(12), 1849-1861.

In any case, in addition to considering additional removal scenarios, motivated by the issue I outline in Major Comment 1 it would be worth repeating the analysis with a slightly different measure of nutritional stability. Currently, the maximum number of nutrients is determined on a network-by-network basis (see Fig. 1). However, this approach does not account for different baseline nutrient provision in different countries and time periods. As such, it would be interesting to repeat the analysis but with a network-independent maximum for the number of nutrients (i.e., nutrient provision) on the y-axis. Nutritional stability could then be given in terms of the area realized compared to the maximum possible area achievable (i.e., consistent with maximum number of nutrients remaining until essentially all crops are removed from a network).

Good idea! Indeed, we went back and forth on this point in earlier versions of the analysis. We initially set an independent maximum (i.e. 17). As you point out, this provides a relativized metric based on "area realized" where the nutritional stabilities of countries are now relative to a global, network-independent maxima. We argue for continuing to present network-dependent, 'absolute' nutritional stability values for the following reasons:

1. As you point out above, a network-independent metric would be valuable *if there is substantial variation "between countries and between time points in terms of nutrient provision".* However, we do not see substantial variation in the # of nutrients per network: the mean ± SD of # of nutrients is 16.4 ± 2.0 (with 90% of all networks having >= 16 nutrients). See Fig. S2 above.

2. Therefore, when we correlate the different approaches to measuring nutritional stability (network-dependent v. network-independent) for 2016 crop-nutrient networks we find they are closely related (R^2 = 0.99 for both production scenarios, see below).

3. Finally, in deriving R_N on a network-by-network basis we keep stability values 'on their own scale'. We believe this is useful if we are interested in knowing what the absolute nutritional stability of a crop nutrient network is based on the existing set of available foods, as we are here.

We believe there is value in both these approaches. For the reasons above (particularly the results from #2), we believe we are justified in keeping our metric as is.

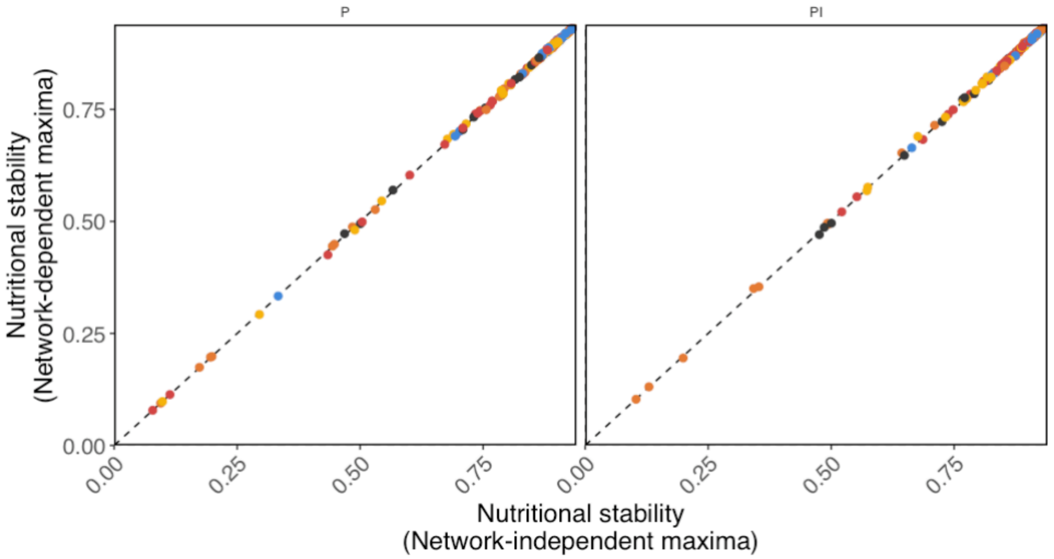

Comparing two approaches to measure nutritional stability (R_N). We calculated R_N with the maximum number in its network ("dependent maxima") and a network-independent maxima (i.e. 17; "independent maxima") for 2016 crop-nutrient networks. The 'line of equality' is dashed. The R^2 for both relationships is 0.99.

In fact, this additional analysis motivates a number of questions that, surprisingly, are not mentioned in the manuscript: What is the optimum crop portfolio for a country to hold? What is the similarity in crop portfolios (and nutrient availability) among countries?

These are interesting questions, thank you for raising them. To your first point, this is something that we also initially discussed. Our approach could permit the identification of optimally stable crop portfolios. However, we chose not to identify "optimum crop portfolios" because we were concerned this approach would be overtly prescriptive. There are numerous economic and cultural factors that would make defining an "optimal portfolio" for a country problematic. Instead we stick to reporting results of past and existing portfolios. In future analyses, we hope to further understand how crop diversity links to nutritional outcomes, which would provide a much greater basis for making claims about optimal numbers of crop portfolios, since we could more clearly link these outcomes to nutrition indicators.

To your second point, this work (similarity in crop portfolios between countries) has already been addressed by Khoury et al. 2014 using the same FAO data. Although those authors do not test for similarity between nutrients, we expect that there is substantial similarity given that there are 17 different nutrients and the average number of nutrients per region for different scenarios is quite similar (see above).

My main point is that nutritional stability on its own may not tell the whole story.

Yes, we agree! We do not ever claim that nutritional stability perfectly characterizes a food system. Instead this is another tool that can help identify system-level attributes of food systems. Just as nutritional status cannot be determined from dietary intake alone, nutritional stability cannot alone determine the availability, let alone utilization, of nutrients. As reviewer 1 points out, and as we discuss (L261-268), there are many aspects to understanding the nutritional ecology of a food system. We have included a caveat in the Methods that further describes what our nutritional stability metric does not do (L372-375):

“Our unitless measure represents how robust a food system is to the sequential elimination of crops. It does not provide information on the identity of different crops or nutrients, nor the utilization of nutrients or the nutrient adequacy of a selection of foods.’

And in the Discussion (L208-213):

“To be clear, we are measuring the relationships of crop diversity to nutrients and their susceptibility to disturbance; we are not measuring nutritional outcomes such as dietary intake, dietary diversity, or other health-related outcomes that are the result of nutrition. Just as nutritional status cannot be determined from dietary intake alone, nutritional stability does not determine the availability, let alone utilization, of nutrients. This is however, a natural area to expand this work moving forward.”

(3) Discuss network analysis limitations

The manuscript includes much discussion (P8 to P11) of the implications of their main finding (that nutritional stability increases then saturates with increasing crop diversity) but less about the limitations of their approach, which should be addressed.

We have now added further discussion of methodological limitations to our caveats section.

The effects of not including relative crop abundances should be discussed. Right now, the probability of losing a crop is the same for all crops, but this is hardly realistic. How much would results be affected if, for example, less abundant crops were lost first? It would also be better to consider the effect of a fractional loss of crops on nutrient provision, rather than the current, "all or nothing" approach. It is worth noting that these considerations---probabilistic loss and fractional loss---are implemented in the Bayesian network approach (Eklöf et al. 2013).

Again, an astute point. We now discuss the limitations of using qualitative (based on presence/absence) rather than quantitative networks (based on abundance, amount, etc.) at L268-275:

“There are also some methodological limitations. Crops are likely to vary in their loss susceptibility according to exogenous factors, such as market value or climate change vulnerability or pest pressure or simply abundance. In our current approach, all crops have equal removal probability; crop removal scenarios that account for these differential vulnerabilities is an exciting next step. Considering fractional crop loss, rather than the current “all or nothing” approach, could also add realistic complexity. Furthermore, complex system modeling of trade dynamics could explore to what extent import-based network re-orientation (i.e., permit crop substitutability) rescues nutritional stability.”

This is again a point where we strive to balance complexity and comprehensibility. Our aim with this initial work was to present our conceptual approach and describe current and historic distributions of nutritional stability between countries. Although the random (i.e. equally probable) loss approach we use is parsimonious, we believe that there is great potential in next steps to consider “informed” extinction loss. As you point out above, crops are likely to vary in loss susceptibility according to other exogenous factors, such as market value or climate change vulnerability or pest pressure or simply abundance. We plan to explore these scenarios in future work. However, we believe that given the complexity of creating the data that underpins many of these “informed loss” scenarios (e.g. climate risk values for all FAO crop types) it is beyond the scope of the present work to tackle these interesting questions.

Network rewiring (e.g., Staniczenko et al. 2010; Vizentin-Bugoni et al. 2020) should also be discussed, both in terms of previous work and next steps. While rewiring in the crop-nutrient networks would be different from in ecological networks (a predator instigates a new trophic interaction if it loses a prey item), it is still likely to happen. For example, it is unlikely a country will do nothing as it loses all its crops, but would instead begin to import comparable crops to fill the nutritional deficit. It is worth noting that, in addition to implementations of rewiring in the authors' current approach (see Staniczenko et al. 2010 and Vizentin-Bugoni et al. 2020 for examples), a method for rewiring has been proposed in the Bayesian network approach (Baldock et al. 2019).

Despite often thinking about re-wiring in ecological contexts, we hadn’t really considered this phenomenon here. Probably because the dynamics behind topological shifts are quite different in crop-nutrient networks when compared to ecological communities. As you point out, the concept of rewiring, while intuitive for predator-prey or plant-pollinator networks, is somewhat different for crop-nutrient networks. Unlike a plant-pollinator network, nutrients are intrinsic to the crop and therefore their link topology cannot be shifted. A crop cannot suddenly provide a nutritional link to Vitamin A, in the way a bee could shift visitation to a different plant. However, we agree that the scenario that you describe (different crops could be added to a network) is completely likely. We now discuss the potential for network ‘re-orientation’ on L273-275. We agree that future work

should explore the potential for network re-wiring/orientation responses to crop loss and its effect on nutritional stability.

MINOR COMMENTS

-- Include in the main text a brief description of the methods workflow and an interpretation of R_N.

We had something of this sort in earlier versions but omitted it for concision. We now state (L65-68):

“Our analytical framework (Fig. 1) links crops and their constituent nutrients into a bipartite network and then quantifies the effect on nutrient availability in a given country when crops are removed from the network, yielding a unitless metric that captures the robustness of different crops in providing nutrients.”

-- P7. Sentence beginning, "Stated succinctly..." It is worth noting that adding low-degree crops may still be worthwhile if they are linked to "vulnerable" nutrients that may only be provided by one or two existing crops (and see comment directly below).

A good point. We have added the following text to make this point (L169-170):

“Nonetheless, it’s worth noting that adding low degree crops may be worthwhile if they provide links to "vulnerable" nutrients – those nutrients with few other links in the network.”

*-- P7. "...stability depends on the number of link within this network." Stability also depends on the *pattern* of connections in the network (otherwise, there would be no need to perform a network analysis and node removal simulations).*

Very true. We now say as much (L187).

-- P12. How many crops are considered in total? 225?

Correct. We know say as much in the Introduction and Methods.

-- P14. How many runs were conducted for each network before averaging?

We ran each network through 1000 permutations. We now say as much (L367).

-- P14. How was the "average attack tolerance" calculated exactly? (Note that this terminology is unconventional: "attack" is typically used to reference removing high-degree nodes first, whereas "error" is typically used to reference random removal.)

We now provide more detail on exactly how we calculated R_N (L366-370):

“At each step k=1,2,...,M the height of the average curve at step k is the average of all the individual curve heights at step k. Finally, we normalize this curve so that max(|$nbr(C^k)$|) = 1 and max(k) = 1 before finding the area under the curve for R_N.”

Also, thanks for this input. We’ve replaced all use of attack tolerance, with “robustness curve” throughout the manuscript

-- P15. Give more details on the autoregressive model. How does it connect to the R_N variance shown in Fig 3B?

We have provided more information on the AR model term (L394-398):

“…and a first order autoregressive (AR-1) correlation structure to account for the temporal dependency structure of our data for each country and scenario (corAR1(form = ~ year|country/scenario). This term assumes correlations between any two years are simply the product of correlations between adjacent years – that is, constant exponential decay of correlation over time.”

The variance depicted in Fig. 3B are not model estimates, rather they are the actual variance in RN over all years for each country.

-- P22. Consider including in Fig. 2 separate panels associated with different time periods, e.g., 1990 to 2000.

We have tried plotting separate panels for different time periods. The general non-monotonic relationships holds over the periods, so this doesn’t add much to the narrative. Moreover, it is unclear when cutoff points for different periods should be and how these should be determined. This is already a panel-dense manuscript, and we think keeping figure 2 as a single panel is best.

-- P23. How are the continental values in Panel 3 calculated?

These values are averages or variances for each country across all years. We now include this information in the figure caption

-- P25. What is the numerical relationship for the linear regression?

We have added a supplemental table (Table S7) that provides the model estimates for this linear regression. We hope this is what you were looking for.

Table S7. Change in crop diversity, degree and nutritional stability. Results are from a linear model testing whether change in crop diversity and degree explain variation in nutritional stability change (R_N ~ diversity change + degree change).

	**Estimate**	**Std. Error**	**t value**	**P value**
Intercept	-0.016	0.005	-3.290	0.001
Crop degree change	0.031	0.002	15.280	<0.001
Crop diversity change	0.010	0.001	16.740	<0.001

Reviewers' Comments:

Reviewer #2:
Remarks to the Author:
Review of revised "Global relationships between crop diversity and nutritional stability" by Nicholson et al.

I am Reviewer 2 from the first round of reviews. I have read the revised manuscript and authors' response letter. I appreciate the authors' response to my comments and find that their edits and additions have improved the manuscript. The extra analyses is welcome and the Supplementary Materials is an appropriate place for their inclusion. When they have chosen not to implement some recommendations, the authors offer arguments that I find reasonable. The extra discussion is helpful, however I recommend the authors include some additional references that would be helpful to readers. Specifically, when covering the Bayesian network approach (L339) they should add references to Baldock et al. 2019 and Haussler et al. 2020; and when covering network rewiring/re-orientation (L246) they should highlight some of the extensive work on this topic already established in ecology such as Staniczenko et al. 2010 and Vizentin-Bugoni et al. 2020. Following these additions, I would be supportive of publication in Nature Communications.

REFERENCES

Baldock, K.C.R. et al. (2019). A systems approach reveals urban pollinator hotspots and conservation opportunities. Nat. Ecol. Evol., 3, 363–373.

Haussler, J., Barabas, G., & Eklof, A. (2020). A Bayesian network approach to trophic metacommunities shows that habitat loss accelerates top species extinctions. Ecology letters, 23, 1849--1861.

Staniczenko, P.P.A., Lewis, O.T., Jones, N.S. & Reed-Tsochas, F. (2010). Structural dynamics and robustness of food webs. Ecol. Lett., 13, 891--899.

Vizentin-Bugoni et al. (2020). Including rewiring in the estimation of the robustness of mutualistic networks. Methods Ecol. Evol., 11, 106--116.

Reviewer #3:
Remarks to the Author:
Review of revision of manuscript revision
NCOMMS-20-43503A
Global relationships between crop diversity and nutritional stability

The authors apply a network analysis to food balance data and thereby provide new insights into the relationship between crop diversity and nutritional stability at national and regional level. The manuscript is intriguing because it triggers important questions on nutritional stability and the role of crop diversity in production and trade thereof. The authors thoughtfully addressed the previous round of reviews and more carefully explain some of the interpretations as well as limitations of the approach.

Coming in as a new reviewer in this second round of review, I have some additional concerns and questions that I consider important to address to add to the clarity and robustness of the approach:

1. In terms of the data used:
• The authors refer to the FAOSTAT food balance data, and this for the 1961 – 2016 timeframe, and thereby refer to crop yield as hg/ha. This is confusing because 1) while FAOSTAT production data provide data on yield expressed as hg/ha, the FAOSTAT food balance data does not provide yield data, but overall amounts produced per country or per capita. 2) The time series of the FAOSTAT food balance data, using the old methodology, go from 1961-2013 (not 2016), and then

the new methodology goes from 2014-2018. So there appear to be some discrepancies but it seems to me that the authors used the production data of FAOSTAT, not the food balance data, and should then also be careful in referring to it as 'available for human consumption'. Please correct me if I am wrong, and where one can then find the hg/ha for the 1961-2016 period in the food balance section of FAOSTAT.

• It is not clear why the authors use yield, in terms of hg/ha for this analysis for this analysis – and I find it confusing to use here hg/ha available for human consumption. Yield does not tell how much is available in total or per capita. Is this just used as a binary variable if this food item is produced or not?

• In terms of the use of agricultural production and import data. I would recommend to not use the term 'scenarios' in this sense. Scenarios is more about different potential paths, while here it is considering different parts of the basket, so I would refer to those as different parts of the food supply. Also it is not clear to me if in terms of the production, you distract what is going for export as it seems you are not using the food balance data but rather the production and trade data.

• Animal-based products are not included. The authors mention this in the methodology but do not emphasize or explain how this limitation reduces the interpretation of the results since animal-based products play a critical role in providing nutrients as well as a buffer/ stock in terms of stability. Coming to the comparison of the shopping basket, it would to only consider part of what is in the basket. This should be more clearly emphasized as a major limitation and therefore the interpretation of nutritional stability of the different regions should also be done with this important limitation in mind – I suspect that the variability between countries will be larger and that trends over time will also differ when taking into account animal-based foods – certainly given the increase of animal-based food production and trade over the last 30 years, considered as a major driver of change in food systems. I would recommend to also include animal-based products in the approach, certainly in the future.

2. In terms of the binary approach for the nutrients in the network analysis:

• This is the part of the methodology I struggle most with: in nutrition it is not only important if the nutrient is there or not but also how much of a nutrient is available and the fluctuation of that availability and this compared to human needs for a healthy diet. In other nutrient approaches this has mostly been taken into account (e.g. Defries et al. 2015; Remans et al 2011, Remans et al 2014). The threshold setting also seems not based on how much of a certain nutrient is actually relevant from a human diet perspective. I suspect that this binary approach towards presence/absence of nutrients in a certain crop leads to an overestimation of nutritional stability as well as to missing important variability between countries. I therefore want to push the authors to reflect more carefully about the limitation of this binary approach in terms of nutrients and include this at least more explicit in the discussion.

3. While I like the overall concept and idea of applying network analysis, I would appreciate some of the critical reflections of the authors of how this could be applied and interpreted to help food system diagnosis, policies and actions

4. The authors use agricultural production and food systems a bit intertwined. They acknowledge that these are not the same but it will be good to reference some literature about food system resilience and discuss how this contributes to this field. This seems part of the literature that this paper aims to add to but doesn't refer much to. Some examples here:

• Bullock, James M., Kiran L. Dhanjal-Adams, Alice Milne, Tom H. Oliver, Lindsay C. Todman, Andrew P. Whitmore, and Richard F. Pywell. 2017. "Resilience and Food Security: Rethinking an Ecological Concept." The Journal of Ecology 105 (4): 880–84.

• Mock, N., C. Béné, M. Constas, and T. Frankenberger. 2015. "Systems Analysis in the Context of Resilience." Technical Series Paper No. 6. Resilience Measurement Technical Working Group. Rome: Food Security Information Network. http://dx.doi.org/.

• Béné, Christophe. 2020. "Resilience of Local Food Systems and Links to Food Security - A Review of Some Important Concepts in the Context of COVID-19 and Other Shocks." Food Security 12 (4): 1–18.

• Understanding the relationships between global supply chain risk and supply chain resilience: the role of mitigating strategies. Juneho Um, Neungho Han. Supply Chain Management. ISSN: 1359-8546. Article publication date: 7 December 2020

REVIEWER COMMENTS

Reviewer #2 (Remarks to the Author):

Review of revised "Global relationships between crop diversity and nutritional stability" by Nicholson et al.

I am Reviewer 2 from the first round of reviews. I have read the revised manuscript and authors' response letter. I appreciate the authors' response to my comments and find that their edits and additions have improved the manuscript. The extra analyses is welcome and the Supplementary Materials is an appropriate place for their inclusion. When they have chosen not to implement some recommendations, the authors offer arguments that I find reasonable.

We again thank you for your constructive and insightful comments. We believe that the MS and analysis is much improved based on your previous comments.

The extra discussion is helpful, however I recommend the authors include some additional references that would be helpful to readers. Specifically, when covering the Bayesian network approach (L339) they should add references to Baldock et al. 2019 and Haussler et al. 2020; and when covering network rewiring/re-orientation (L246) they should highlight some of the extensive work on this topic already established in ecology such as Staniczenko et al. 2010 and Vizentin-Bugoni et al. 2020.

Agreed. We have added the recommended references.

Following these additions, I would be supportive of publication in Nature Communications.

Thank you

REFERENCES

Baldock, K.C.R. et al. (2019). A systems approach reveals urban pollinator hotspots and conservation opportunities. Nat. Ecol. Evol., 3, 363–373.

Haussler, J., Barabas, G., & Eklof, A. (2020). A Bayesian network approach to trophic metacommunities shows that habitat loss accelerates top species extinctions. Ecology letters, 23, 1849--1861.

Staniczenko, P.P.A., Lewis, O.T., Jones, N.S. & Reed-Tsochas, F. (2010). Structural dynamics and robustness of food webs. Ecol. Lett., 13, 891--899.

Vizentin-Bugoni et al. (2020). Including rewiring in the estimation of the robustness of mutualistic networks. Methods Ecol. Evol., 11, 106--116.

Reviewer #3 (Remarks to the Author):

The authors apply a network analysis to food balance data and thereby provide new insights into the relationship between crop diversity and nutritional stability at national and regional level. The manuscript is intriguing because it triggers important questions on nutritional stability and the role of crop diversity in production and trade thereof. The authors thoughtfully addressed the previous round of reviews and more carefully explain some of the interpretations as well as limitations of the approach.

> *Thank you for taking the time to read our manuscript and our previous round of reviews.*

Coming in as a new reviewer in this second round of review, I have some additional concerns and questions that I consider important to address to add to the clarity and robustness of the approach:

1. In terms of the data used:

• The authors refer to the FAOSTAT food balance data, and this for the 1961 – 2016 timeframe, and thereby refer to crop yield as hg/ha. This is confusing because 1) while FAOSTAT production data provide data on yield expressed as hg/ha, the FAOSTAT food balance data does not provide yield data, but overall amounts produced per country or per capita. 2) The time series of the FAOSTAT food balance data, using the old methodology, go from 1961-2013 (not 2016), and then the new methodology goes from 2014-2018. So there appear to be some discrepancies but it seems to me that the authors used the production data of FAOSTAT, not the food balance data, and should then also be careful in referring to it as 'available for human consumption'. Please correct me if I am wrong, and where one can then find the hg/ha for the 1961-2016 period in the food balance section of FAOSTAT.

> *You are correct we used the production data, not the food balance data. Thank you for catching this mistake. We have removed mention of 'food balance data' and are careful in referring to food 'available for human consumption'.*

• It is not clear why the authors use yield, in terms of hg/ha for this analysis for this analysis – and I find it confusing to use here hg/ha available for human consumption. Yield does not tell how much is available in total or per capita. Is this just used as a binary variable if this food item is produced or not?

> *This is a typo. We used the FAO production quantity data (i.e. tonnes). As you point out, the production data is largely used as a binary variable to determine crop presence/absence. We use the amount produced in our thresholding approach to overcome the minimum quantitates problem, where we remove binary network links resulting from crops contributing minimal amounts of certain nutrients (L299-315).*

• In terms of the use of agricultural production and import data. I would recommend to not use the term ‘scenarios’ in this sense. Scenarios is more about different potential paths, while here it is considering different parts of the basket, so I would refer to those as different parts of the food supply. Also it is not clear to me if in terms of the production, you distract what is going for export as it seems you are not using the food balance data but rather the production and trade data.

Good point. We have replaced our use of scenarios with ‘supply source’ throughout the manuscript and supplemental materials. In the present study we do not evaluate exports and only consider the food available in a country from production and production plus imports.

• Animal-based products are not included. The authors mention this in the methodology but do not emphasize or explain how this limitation reduces the interpretation of the results since animal-based products play a critical role in providing nutrients as well as a buffer/ stock in terms of stability. Coming to the comparison of the shopping basket, it would to only consider part of what is in the basket. This should be more clearly emphasized as a major limitation and therefore the interpretation of nutritional stability of the different regions should also be done with this important limitation in mind – I suspect that the variability between countries will be larger and that trends over time will also differ when taking into account animal-based foods – certainly given the increase of animal-based food production and trade over the last 30 years, considered as a major driver of change in food systems. I would recommend to also include animal-based products in the approach, certainly in the future.

Thank you for pushing us to bring this limitation more to the fore. We have referenced ‘nutrients from crops’ at points in the results and discussion to re-emphasize that we are evaluating effects of crop diversity. Moreover, we have added an additional caveat, discussing the good points you raise (L276-282):

“Adequate food and nutritional security comprises much more than the factors captured in our analysis, which provides a relative measure of nutrient availability not an absolute metric of adequacy. In the present study we focused on nutrients available from crops, because animal based products are rarely-resolved to the species level and there is large interspecies variability in crop micronutrient composition. Animal-based products nonetheless play a critical role in providing some nutrients, thus there may be greater variability between countries when accounting for animal-based foods.”

2. In terms of the binary approach for the nutrients in the network analysis:

• This is the part of the methodology I struggle most with: in nutrition it is not only important if the nutrient is there or not but also how much of a nutrient is available and the fluctuation of that availability and this compared to human needs for a healthy diet. In other nutrient approaches this has mostly been taken into account (e.g. Defries et al.

2015; Remans et al 2011, Remans et al 2014). The threshold setting also seems not based on how much of a certain nutrient is actually relevant from a human diet perspective. I suspect that this binary approach towards presence/absence of nutrients in a certain crop leads to an overestimation of nutritional stability as well as to missing important variability between countries. I therefore want to push the authors to reflect more carefully about the limitation of this binary approach in terms of nutrients and include this at least more explicit in the discussion.

This is an excellent point. We acknowledge that the binary (also known as qualitative network) approach is a current limitation of this work. We agree that future work should take into account how much of the nutrient is available in the network and generate removal scenarios based on this. In the present work we largely wanted to demonstrate how methods from network science could be applied to evaluate food system stability (as opposed to availability metrics by Remans, DeFries and others). This is an important distinction. As we state throughout the MS we are not providing a metric of nutritional adequacy, rather a systems-level measure of robustness to disturbance. However, we recognize that the vulnerability of nutrient provision will depend on how much of that nutrient is produced. We are working towards a quantitative network approach, but it is still under development and beyond the scope of the current paper. Nonetheless, we now make more explicit the limitations of our current binary approach (L287-290):

"Our current approach considers only nutrient presence or absence, and may underestimate nutritional stability because ultimately the vulnerability of nutrient provision will also depend on how much of that nutrient is produced. Considering fractional crop loss or removal probabilities based on production levels could add realistic complexity in future analyses"

NB. A binary, "all-or-nothing" approach would tend to underestimate network robustness

3. While I like the overall concept and idea of applying network analysis, I would appreciate some of the critical reflections of the authors of how this could be applied and interpreted to help food system diagnosis, policies and actions.

An excellent suggestion. We have reflected on how the application of this approach could be used (L263-269):

"Scenario development using our metric could help target country-specific crop additions that would maximize nutritional stability. Our approach could also be used to identify potential tradeoffs in production and import outcomes, at least as it relates to the availability of a given amount of nutrients in a certain place. In the context of policy interventions, this system-level metric could be applied in panel-type designs to diagnose whether initiatives (e.g. promoting or increasing food production, trade and storage) at different scales of organization (e.g. household, community, national)

effectively supported food system resilience goals (Mock et al. 2015)."

4. The authors use agricultural production and food systems a bit intertwined. They acknowledge that these are not the same but it will be good to reference some literature about food system resilience and discuss how this contributes to this field. This seems part of the literature that this paper aims to add to but doesn't refer much to. Some examples here:

Bullock, James M., Kiran L. Dhanjal-Adams, Alice Milne, Tom H. Oliver, Lindsay C. Todman, Andrew P. Whitmore, and Richard F. Pywell. 2017. "Resilience and Food Security: Rethinking an Ecological Concept." The Journal of Ecology 105 (4): 880–84.

Mock, N., C. Béné, M. Constas, and T. Frankenberger. 2015. "Systems Analysis in the Context of Resilience." Technical Series Paper No. 6. Resilience Measurement Technical Working Group. Rome: Food Security Information Network. http://dx.doi.org/.

Béné, Christophe. 2020. "Resilience of Local Food Systems and Links to Food Security - A Review of Some Important Concepts in the Context of COVID-19 and Other Shocks." Food Security 12 (4): 1–18.

Understanding the relationships between global supply chain risk and supply chain resilience: the role of mitigating strategies. Juneho Um, Neungho Han. Supply Chain Management. ISSN: 1359-8546. Article publication date: 7 December 2020

Thank you raising this point. We agree that agricultural production is only one aspect of a food security and food systems. We already cite the Bullock et al 2017 paper (reference #11) and Béné et al's earlier (2016), and perhaps more general paper on food system resilience (reference #17). We have added Mock et al in our discussion of applying this metric (see above) as well as the defining work by Tendall et al. 2015. Unfortunately, the article by Um & Han is behind a paywall and could not be accessed. We now discuss how our work fits into the field of food system resilience (L212-215):

*"*By considering both production and nutritional diversity our work advances the quantification of food system resilience – the capacity over time of a food system and its units at multiple levels, to provide sufficient, appropriate and accessible food to all, in the face of various and even unforeseen disturbances (Tendall et al. 2015). Our results have many important implications for our understanding of nutritional measures and their relationship to crop diversity. First, our work reaffirms the existing body of research demonstrating that crop diversity is important for agricultural resilience (Bullock et al. 2017), and it does so at a national scale. Much of the existing work in understanding crop diversity and its links to nutritional outcomes are at a field or landscape level (Tobin et al. 2019)."

Reviewers' Comments:

Reviewer #3:
Remarks to the Author:
The authors have addressed most comments efficiently.

Two remaining comments to be addressed:

1. The added paragraph on the justification for not including animal source foods in inadequate. The authors note `In the present study we focused on nutrients available from crops, because animal based products are rarely-resolved to the species level and there is large interspecies variability in crop micronutrient composition.' This is a very weak justification. Animal-based products can be resolved at species level based on FAOSTAT data, this has been done it other cases, it is just a bit more work. Further there is also a lot of interspecies variability in animal-based food sources. I therefore suggest that the authors contextualize their decision for not including animal-based sources more like something as follows: This study pioneers nutritional stability as a method and concept using crop data. Animal-based products can also play an important role in providing nutrients and for stability in case of shocks and anomalies. The role of animal-sourced products is beyond the scope of this paper but will be of interest to investigate in further research.

2. The authors note that 'Much of the existing work in understanding crop diversity and its links to nutritional outcomes are at a field or landscape level.'

There are studies that have looked at global and national level to these relationships - these should be recognized - see for example

Remans et al. 2014
https://www.bioversityinternational.org/fileadmin/user_upload/online_library/publications/pdfs/Measuring_nutritional_diversity_of_national_food_supplies_1951.pdf,

and also Herrero et al. 2017
https://pubmed.ncbi.nlm.nih.gov/28670647/
among others.

I also recommend to change in this sentence the term 'nutritional outcomes' to 'nutritional variables' since this study does not look at human/ population based nutritional outcomes such as stunting, wasting, underweight, obesity, NCDs, anemia.. but at nutrition-relevant variables.

Reviewer #3 (Remarks to the Author):

The authors have addressed most comments efficiently.

Two remaining comments to be addressed:

1. The added paragraph on the justification for not including animal source foods in inadequate. The authors note `In the present study we focused on nutrients available from crops, because animal based products are rarely-resolved to the species level and there is large interspecies variability in crop micronutrient composition.' This is a very weak justification. Animal-based products can be resolved at species level based on FAOSTAT data, this has been done it other cases, it is just a bit more work. Further there is also a lot of interspecies variability in animal-based food sources. I therefore suggest that the authors contextualize their decision for not including animal-based sources more like something as follows: This study pioneers nutritional stability as a method and concept using crop data. Animal-based products can also play an important role in providing nutrients and for stability in case of shocks and anomalies. The role of animal-sourced products is beyond the scope of this paper but will be of interest to investigate in further research.

Thank you for this suggestion. We have included the rationale you provide.

2. The authors note that 'Much of the existing work in understanding crop diversity and its links to nutritional outcomes are at a field or landscape level.' There are studies that have looked at global and national level to these relationships - these should be recognized - see for example

Remans et al.
2014 https://www.bioversityinternational.org/fileadmin/user_upload/online_library/publications/pdfs/Measuring_nutritional_diversity_of_national_food_supplies_1951.pdf,

and also Herrero et al. 2017
https://pubmed.ncbi.nlm.nih.gov/28670647/
among others.

Thank you for bringing these to our attention. Remans et al. 2014 is foundational to the present work and already cited in the Introduction. We have revised this sentence to recognize these works. We now say: "Previous work has examined patterns of crop or nutritional diversity at global scales[15,28], or linked crop diversity and nutritional variables at field or landscape levels [9]."

I also recommend to change in this sentence the term 'nutritional outcomes' to 'nutritional variables' since this study does not look at human/ population based nutritional outcomes such as stunting, wasting, underweight, obesity, NCDs, anemia.. but at nutrition-relevant variables.

Noted. We have changed this to 'nutritional variables'
